# Hydrothermal Humification of Biomass for Circular Carbon Management in Sustainable Agro‐Ecosystems

**DOI:** 10.1002/advs.75558

**Published:** 2026-06-23

**Authors:** Ziyun Liu, Zonglu Yao, Yuanhui Zhang, Huiyan Zhang, Lixin Zhao

**Affiliations:** ^1^ Key Laboratory of Low‐carbon Green Agriculture in North China Ministry of Agriculture and Rural Affairs Institute of Environment and Sustainable Development in Agriculture Chinese Academy of Agricultural Sciences Beijing P. R. China; ^2^ Department of Agricultural and Biological Engineering University of Illinois at Urbana‐Champaign Urbana Illinois USA; ^3^ School of Energy and Environment Southeast University Nanjing P. R. China

**Keywords:** artificial humic substances, biomass waste valorization, hydrothermal humification, soil carbon sequestration, sustainable agriculture

## Abstract

Hydrothermal humification (HTH) is emerging as a low‐carbon approach for valorizing biomass into hydrothermal humic acid (HHA), a multifunctional soil amendment with potential to enhance soil carbon sequestration and climate‐resilient agriculture. However, broader deployment is constrained by limited carbon‐conversion efficiency and heterogeneity in HHA composition. This Review synthesizes recent advances in HTH process engineering, elucidating how operating conditions, catalytic strategies, and integrated designs govern HHA yield, molecular structure, reactivity, and stability. Comparative analysis of hydrolysis‐, condensation‐, and oxidation‐dominated pathways reveals distinct carbon‐transformation mechanisms and structure–function relationships underlying soil and plant responses. Life‐cycle and technoeconomic assessments highlight pathways toward environmentally and economically viable deployment. Advancing HHA stability and field‐scale integration are key frontiers for HTH. Progress toward scalable negative‐emissions deployment in sustainable agro‐ecosystems will require close collaboration between scientists and engineers.

## Graphical abstract

This article provides a comprehensive overview of biomass hydrothermal humification for building sustainable agro‐ecosystems. It examines the chemical transformation mechanisms and the functional benefits of the produced humic acids in soil remediation. The review identifies key challenges and future opportunities for large‐scale carbon sequestration, offering a roadmap for achieving carbon neutrality through innovative waste valorization within the circular economy.

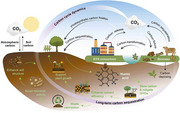

## 1 Introduction

**FIGURE 1:**
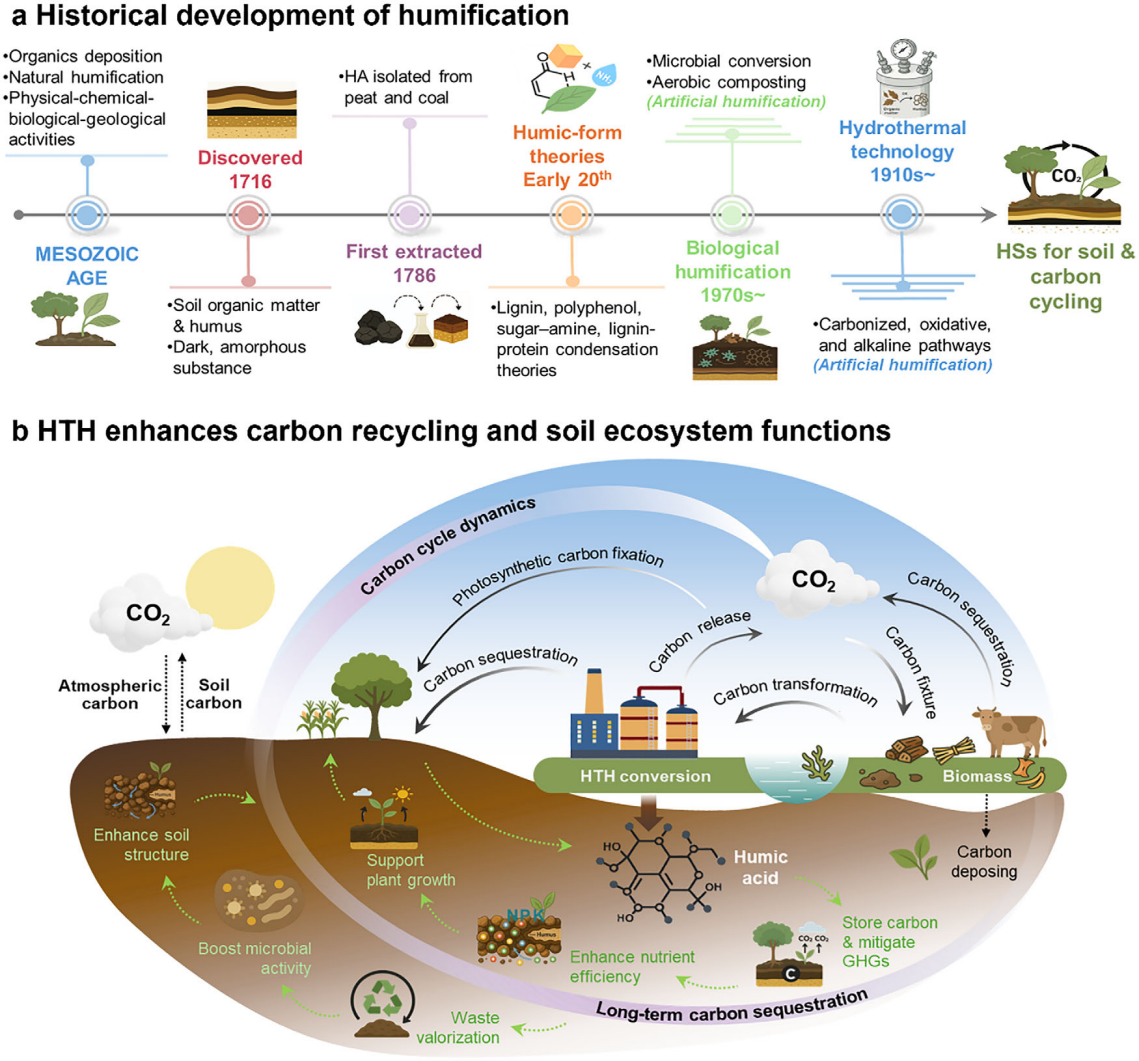
Historical evolution of HSs and HTH roles in soil ecosystem functioning. (a), The historical timeline of HSs research and technological development. (b), Key functions of HHA in enhancing soil fertility, nutrient cycling, plant productivity, and environmental remediation, while contributing to long‐term carbon storage and greenhouse‐gas emissions mitigation within the carbon cycle.

Soil organic matter (SOM), with a global stock estimated at approximately 1500 Gt, is a cornerstone of the global carbon cycle and a critical lever for climate change mitigation [1]. Humic substances (HSs), which account for over 80% of SOM, form one of the largest and most stable terrestrial carbon pools [2] (Figure 1a). The global HSs market is expected to grow by 11.8% annually, reaching USD $1.1 billion by 2028 [3]. Meanwhile, soil organic carbon stocks are declining by 600–700 kg ha^−^^1^ due to intensive farming, fertilizer overuse, and climate change [4]. Sustainable agriculture requires maintaining total carbon above 5 wt.%, with a minimum of 1 wt.% in farmland soils [5]. However, peatlands rich in natural HSs cover less than 3% of the Earth's land [6]. The ongoing depletion of SOM, particularly HSs, poses serious risks to soil health, long‐term agricultural productivity, and global food security.

HSs consist of humic acid (HA), fulvic acid (FA), and humin. HA refers to a dark, high‐molecular‐weight fraction (600–100 000 Da) accounting for 30%–50% of SOM [7]. It enhances soil microbial activity, retains nutrients and water, and promotes plant growth. These functions arise from its aromatic and aliphatic backbones enriched with reactive groups such as carboxyls and quinones [8]. FA has lower molecular weights (600–1 500 Da) that can evolve toward HA‐like structures, whereas humin is an insoluble, highly recalcitrant fraction that underpins long‐term carbon stabilization [8, 9, 10]. Beyond agriculture, HA is increasingly applied in pharmaceuticals, energy storage, and environmental remediation due to its redox activity [3, 11]. However, natural HA forms slowly over centuries through biomass decomposition, and most commercial HA extracted from non‐renewable peat and lignite shows low yields (13.6%–83.8%) and poor sustainability [12, 13], while biological humification of biomass waste produces <10% HA and requires at least a month of processing [14, 15, 16]. Hydrothermal humification (HTH) has emerged as a sustainable, carbon‐neutral technology that mimics natural humification under subcritical water (160°C–300°C) and autogenous pressure, completing within hours. Recognized by the International Union of Pure and Applied Chemistry as one of the “Top 10 Emerging Technologies in Chemistry” in 2021, HTH offers a promising pathway to renewable HSs.

HTH humic acid (HHA) closely resembles natural HA in key functional groups and exhibits comparable or even superior agro‐ecosystem benefits (Figure 1b) [17]. Their main differences lie in formation pathways and feedstock diversity [18]. Natural HA have heterogeneous structures that forms slowly through microbial–chemical interactions and environmental processes, whereas HHA is synthesized under hydrothermal conditions, relying on rapid hydrolysis and polycondensation of organic matter. Compared with natural humification, HTH enables the rapid, controllable production of HHA with simpler, tunable structures. Substantial progress has been made in improving both yield and quality of HHA, with recovery rates reaching approximately 30% of feedstock mass. Catalytic systems and integrated procedure have played a pivotal role in accelerating humification in neutral/acidic, alkaline, and oxidative conditions. HTH is emerging as a promising platform for engineered humification, but the mechanistic controls of carbon transformation, particularly the thermodynamic drivers and molecular evolution pathways (e.g., O/C–H/C trajectories), remain insufficiently understood. In addition, the structure‐property‐function relationships governing long‐term HHA stability and agronomic performance are not yet clearly established, with limited comparative evidence across different HTH routes. Advancing this integrated understanding is essential for predictable and durable carbon storage, and for establishing HTH as a reliable negative‐emissions solution.

In this Review, we present the HTH technological framework, key reaction parameters, carbon‐conversion‐enhancing strategies, and HHA applications in sustainable agro‐ecosystems, along with sustainability and technoeconomic considerations. We begin by outlining the principles and mechanisms of HTH, followed by advanced hydrolysis‐, condensation‐, and oxidation‐dominant strategies that improve humification efficiency and HHA performance. We then discuss HHA functions in carbon retention, soil improvement, and plant‐growth promotion. Finally, we assess the technoeconomic performance and environmental impacts of HTH to evaluate its carbon‐negative potential and prospects for practical deployment.

## 2 A Conceptual Framework on Biomass Humification

**FIGURE 2:**
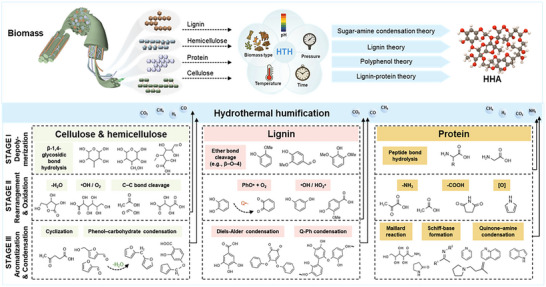
HTH framework showing conversion of biomass components to humic precursors.

Natural humification theories provide a useful framework for interpreting humification reactions under subcritical HTH conditions, and biomass feedstock composition is a primary factor that determines which pathways dominate during HTH (Figure 2). Feedstocks can be broadly categorized into lignocellulosic and non‐lignocellulosic types.

**FIGURE 3:**
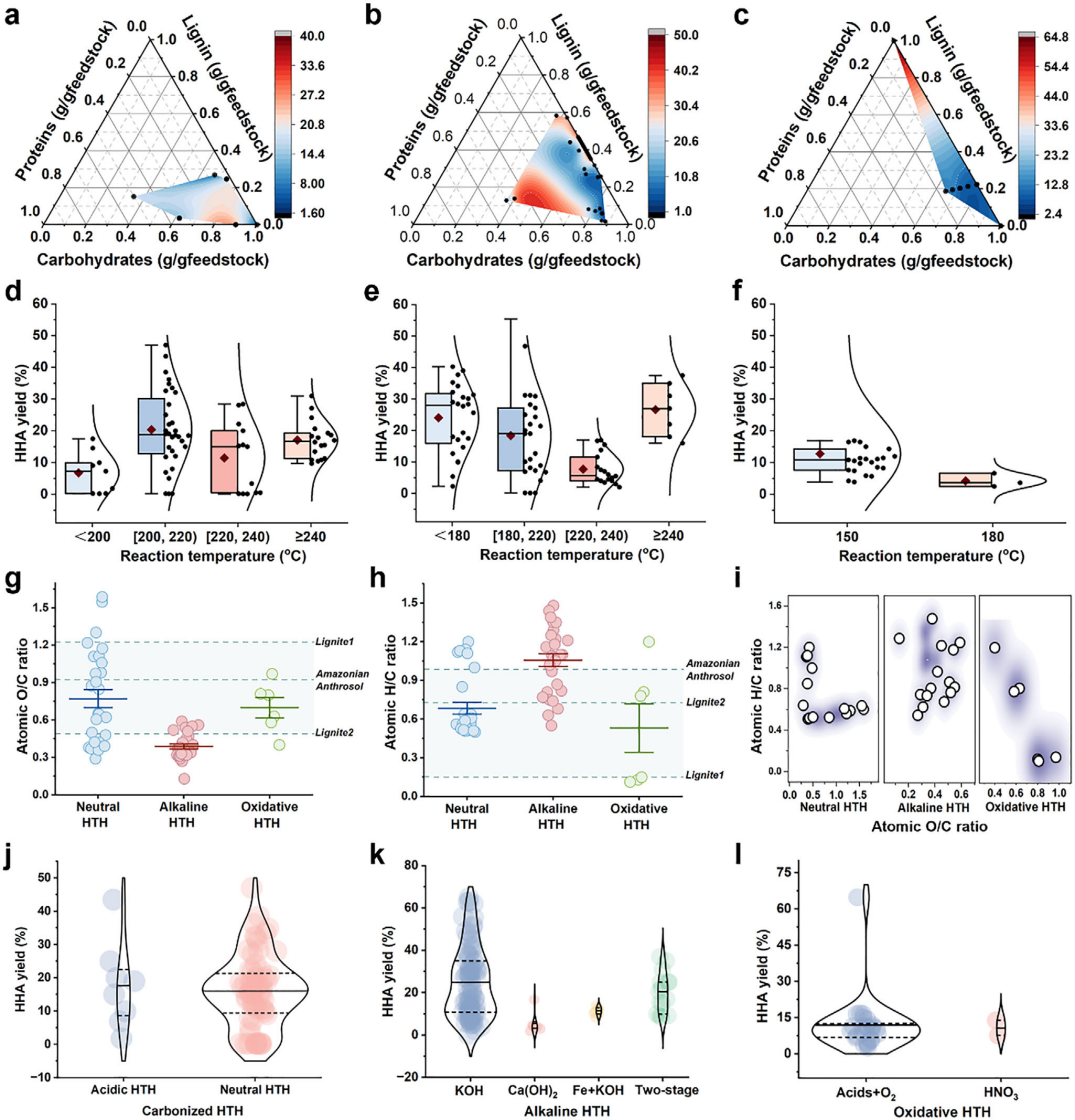
Summary of HHA yield and elemental composition across different HTH processes. (a–c), Ternary contour plots showing the effects of biomass composition (carbohydrates, lignin, and protein) on HHA yield under neutral, alkaline, and oxidative conditions. (d–f), Box plots of HHA yield at varying reaction temperatures across the three HTH pathways. (g–i), Atomic O/C and H/C ratios of HHA products, reflecting degrees of oxidation and aromaticity. (j–l), Violin plots illustrating the distribution of HHA yields under neutral, alkaline, and oxidative processes. Data collected from [12, 18, 19, 23, 24, 25, 26, 27, 28, 29, 30, 31, 32, 33, 34, 35, 36, 37, 38, 39, 40, 41, 42, 43, 44, 45, 46, 47, 48].

For lignocellulosic substrates, lignin is the primary contributor to HHA precursors [19] (Figure 3a–c). In natural humification, the lignin and polyphenol theories describe the depolymerization of lignin into methoxy‐substituted phenolics, followed by oxidation to polyphenols and carboxyl‐rich aromatics [20, 21]. Similar reactions occur during HTH, where lignin‐derived phenolics are rapidly transformed by reactive radicals such as H• and •OH into o‐hydroxyphenols and carboxylic acids [22], whereas pyrolysis favors aromatization and condensation under high‐temperature, oxygen‐limited conditions, producing highly recalcitrant, carbon‐rich biochar. In addition, carbohydrate fractions under hydrothermal conditions, particularly in acidic environment, can undergo dehydration reactions to form furanic compounds such as furfural and 5‐hydroxymethylfurfural (5‐HMF) [23], which may further participate in condensations with lignin‐derived aromatics to generate HHA precursors [19]. These intermediates may therefore play a dual role, serving as both reactive precursors and potential sources of phytotoxicity. Excessive accumulation of partially transformed furanic and phenolic intermediates may raise concerns for agricultural applications [23]. It should be noted, however, that these effects are strongly dependent on reaction conditions and may be mitigated through appropriate optimization strategies. Therefore, careful control of reaction parameters such as temperature, residence time, and acidity together with post‐treatment strategies, is essential to balance precursor formation and by‐product suppression.

Non‐lignocellulosic feedstocks such as food waste, algae, and sewage sludge follow pathways that resemble the lignin–protein and sugar–amine condensation theories [22]. Proteins and lipids supply amino, amide, and carboxyl groups through deamidation, Maillard reactions, and sugar–amine condensation [20, 24]. These reactions incorporate nitrogen‐containing functionalities into HHA and generate oxygen‐rich aromatic precursors such as C_2_H_2_O_2_, C_3_H_4_O_2,_ and C4H6O2 [25, 26]. In some cases, deamidation can account for nearly half of the observed molecular transformations [25]. It is worth noting that in thermally processed biomass (e.g., roasted residues), nitrogen‐containing heterocycles such as pyrazines may form via Maillard‐type reactions and contribute to the nitrogen pool, and are generally more biodegradable and less toxic than pyridine‐type compounds. These natural humification concepts help explain how lignin, carbohydrates, proteins, and lipids interact during HTH to generate HHA with tunable aromaticity, nitrogen content, and functional‐group profiles.

## 3 Triggers of HTH Parameters on HHA Formation

**FIGURE 4:**
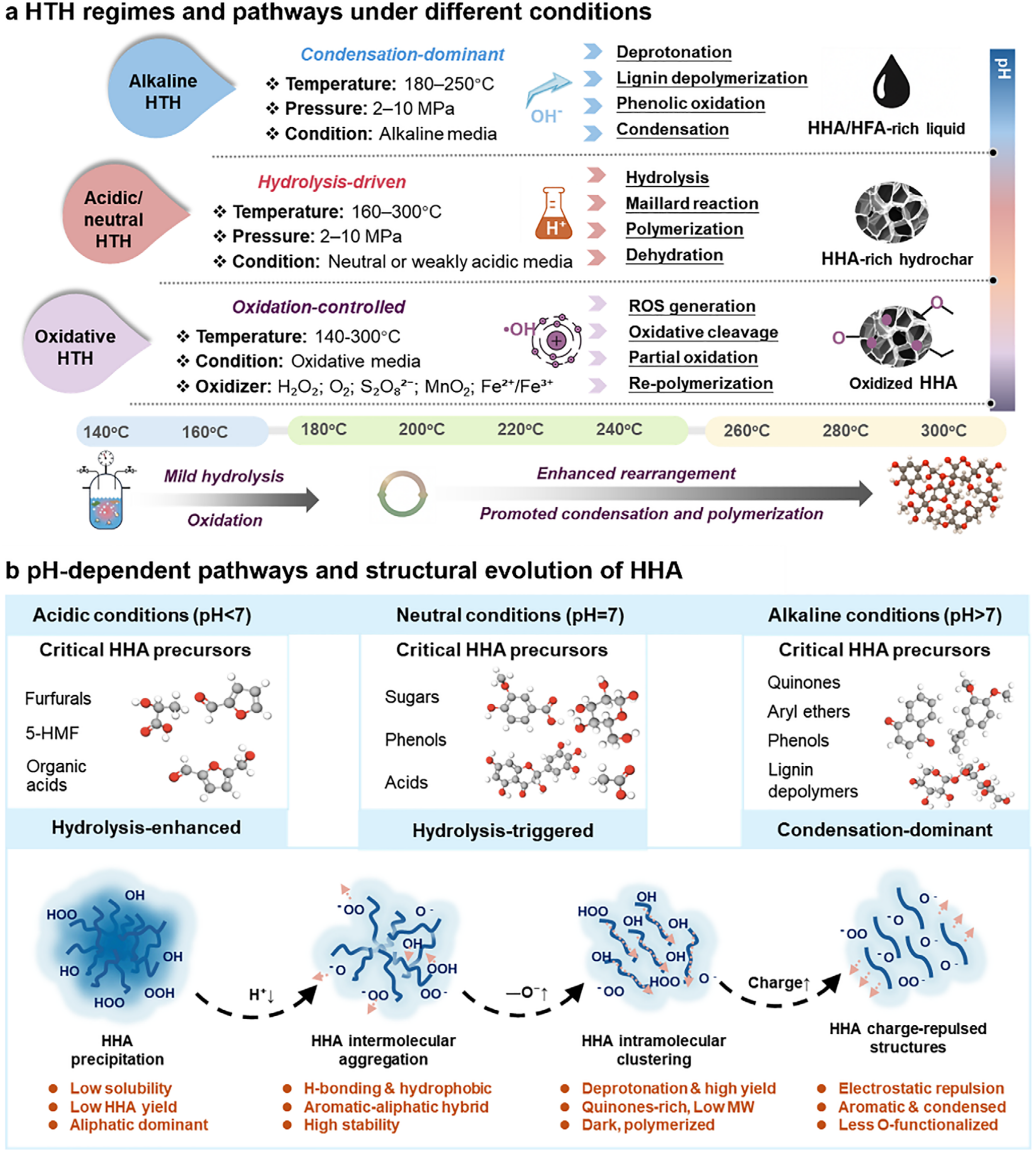
HTH pathways under different conditions. (a), Schematic overview of HTH regimes under different reaction environments, showing shifts in dominant pathways and products. (b), pH‐dependent HHA formation, where alkalinity promotes lignin depolymerization and phenolic condensation, yielding less aggregated HHA structures.

This section examines how key HTH parameters, including temperature, pressure, residence time, pH, and biomass‐to‐water ratio, influence humification efficiency, molecular‐weight distribution, and functional‐group development (Figure 4).

### 3.1 Thermodynamic Process

Temperature is the primary driver of humification during HTH (Figure 3d–f). At 140°C–160°C, lignocellulose, proteins, and lipids hydrolyze into low‐molecular‐weight precursors that subsequently condense into HHA. For corn stalk digestate and Kraft lignin, peak HHA yields occur at 150°C with enriched aromatic and oxygenated structures [26, 27]. Between 160°C and 240°C, humification is generally optimal across diverse feedstocks, such as agroforestry waste [28], vegetable waste [19], lincomycin fermentation residues [49], Chinese medicine residues [29], invasive weeds [30], and food waste [31], with yields increasing from 180°C to 220°C due to intensified formation of carbohydrate‐derived intermediates (Figure 3d,e). Beyond 220°C, yields decline because of excessive cracking and gasification. Higher temperatures enhance hydrolysis and conversion. At 200°C–240°C, lignin depolymerizes extensively, cleaving ether and ester bonds and generating aromatic monomers that initiate HHA formation. Lignin derivatives can polymerize or condense with other intermediates, such as furfural, to form bioactive aromatic compounds (e.g., vanillin and methionine sulfoxide) [32, 33], which generally occur at relatively lower temperatures. Notably, the balance between depolymerization and recondensation is strongly temperature‐dependent: temperatures above the ceiling temperature favor bond cleavage, whereas recondensation processes are more favorable under moderate conditions. HTH and hydrothermal carbonization (HTC) operate over similar temperature ranges but diverge in product formation, with HTH producing humic‐like substances (HHA/HFA) and HTC yielding more carbon‐dense, less reactive hydrochar, accompanied by differences in catalyst, pH, and residence time.

Hydrothermal pressure is generated autogenously and acts synergistically with temperature. Studies with independently controlled pressure show that external pressure accelerates biomass depolymerization and dehydration [50]. HHA selectivity reaches 66.01% at 200°C and 4 MPa, and a maximum yield of 35.28% is achieved at 160°C and 6 MPa [51]. Pressure also promotes the formation of condensed aromatic structures associated with long‐term carbon stability [52]. High‐pressure oxidative conditions further enhance lignin conversion into acidic aromatic precursors [53]. Decoupling pressure and temperature effects remains an important research need. Although higher temperatures and pressures promote HHA formation, their increased energy demand and associated emissions must be carefully balanced.

### 3.2 Kinetic Process

Residence time governs precursor evolution during HTH. At 160°C–220°C, prolonged reaction time promotes condensation, with HHA yields increasing steadily from 1 to 4 h for vegetable wastes and fermentation residues [19]. In contrast, for corn stover digestate [26], Kraft lignin [27], food waste [54], invasive plants [30], and lincomycin fermentation residues [49], maximum HHA yields are typically achieved within 2 h. Below 220°C, insufficient residence time leads to incomplete humification, whereas protein‐rich feedstocks may require extended treatment [19]. For example, wastewater sludge requires up to 12 h at 170°C to reach peak HHA yield, coinciding with enhanced hydrolysis, oxidation, deamination, and ring‐opening reactions [25].

At high temperatures (260°C–300°C), rapid hydrolysis allows HHA formation within a short residence time [25], although prolonged exposure may lead to over‐decomposition or melanoidin formation. Because both time and temperature strongly influence energy consumption, practical operation requires careful optimization supported by technoeconomic analysis.

### 3.3 Proton‐Controlled Process

Humification pathways are highly pH‐dependent (Figure 4b). Under acidic to neutral conditions, hydrolysis and aggregation dominate. Carbohydrates and proteins undergo extensive hydrolysis, while lignin partially depolymerizes, yielding HHA with a higher molecular weight and greater aromatic condensation [55]. The pH typically decreases during the early stage of HTH due to the accumulation of organic acids. Acidic conditions can also enhance nutrient recovery and bioactivity, although excessive acidity favors humin formation over HHA [54, 56].

Under alkaline conditions (pH 9–14), deprotonation and OH^−^‐induced bond cleavage increase lignin solubility and promote phenolic oxidation–condensation [19, 26]. The resulting HHA exhibits lower O/C and higher H/C ratios, indicating more aliphatic and hydrophilic structures (Figure 3g–i). Retro‐aldol and hydrogen‐transfer reactions generate organic acids and furan derivatives [23]. KOH at 5 wt.% provides optimal humification efficiency with limited formation of inhibitory by‐products [26, 34, 49]. Nevertheless, prolonged residence time can lead to humin formation [28]. Two‐stage acid–alkaline strategies and aqueous‐phase recycling further increase HHA selectivity to 65%–72% [57, 58, 59], offering an environmentally compatible route for efficient HHA producing.

### 3.4 Mass Transfer Process

The biomass‐to‐water ratio, expressed as total solids (TS), influences reaction kinetics and product distribution. Higher TS shortens heating time, concentrates intermediates, suppresses excessive liquefaction, and enhances mass retention [34]. Increasing TS from 10% to 20% significantly improves HHA and hydrochar yields while enhancing nutrient retention [34]. Reduced water activity at elevated TS shifts reaction equilibria toward more polymerized and aromatic humic structures, favoring Maillard‐type condensation and nitrogen retention, in accordance with Le Chatelier's principle. Adjusting TS therefore, provides a practical lever to improve HHA yield, stability, and nutrient recovery.

## 4 Carbon‐Conversion‐Enhanced HTH Strategies

The dominant humification pathway in HTH is governed by reaction conditions. Hydrolysis‐, condensation‐, and oxidation‐dominated regimes arise under alkaline, weakly acidic or neutral, and oxidative environments, respectively (Figure 4a). Importantly, these pathways are not discrete but dynamically coupled. Hydrolysis generates reactive intermediates (e.g., sugars, phenolics, and organic acids), which subsequently undergo condensation and oxidation, driving progressive molecular transformation. Although these processes share common reaction routes, differences in intermediate accumulation and reaction priority result in distinct HHA yields and structural properties.

### 4.1 Hydrolysis‐Initiated HTH Strategies

Under neutral to mildly acidic conditions, HTH primarily produces hydrochar composed of HHA and humin. Typically, approximately 50% of the initial biomass is retained as hydrochar, containing 60%–80% of the carbon, while the remainder partitions into liquid and gaseous phases. From this hydrochar, 30%–60% HHA can be extracted (Figure 3j). Humification proceeds rapidly, often within 24 h, mimicking natural humification on an accelerated timescale. Strategies to enhance carbon conversion primarily involve metallic catalysts and acid additives.

**FIGURE 5:**
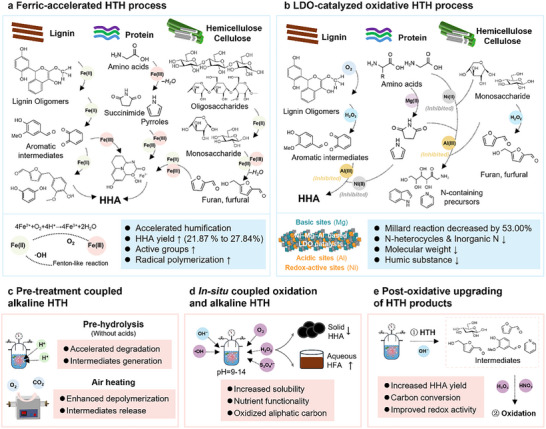
Catalytic HTH mechanisms and integrated humification‐enhancing strategies. (a), Fe^2^^+^/Fe^3^^+^ catalysts enhance biomass depolymerization and condensation to promote HHA formation; Mn^2^^+^ drives redox‐induced degradation and aromatic condensation. (b), Ni–Mg–Al LDO catalysts accelerate hydrolysis via basic sites and suppress Maillard‐type reactions via acidic sites to facilitate HFA formation; H_2_O_2_ and O_2_ further intensify oxidative depolymerization and the generation of functionalized, aromatic‐rich humic structures. (c), Pretreatment–alkaline HTH coupling, including pre‐hydrolysis and air‐heating steps that increase depolymerization rates and humification potential. (d), In situ coupled oxidation and alkaline HTH, where reactive oxygen species accelerate lignin depolymerization, increase carboxylation, and enhance solubility and nutrient functionality. (e), Post‐oxidation of hydrochar or HTH liquors using HNO_3_, H_2_O_2_, or air heating to increase HHA yield, aromaticity, and functional‐group density.

Ferrous salts and oxides (i.e., FeSO_4_, Fe_3_O_4_) promote HHA formation via Fenton‐like generation of hydroxyl radicals (•OH), which cleave lignocellulosic C─C and C─O bonds and generate reactive intermediates such as furfural, 5‐HMF, and phenolics (Figure 5a). For example, the addition of FeSO_4_ increases HHA yields from 21.87% to 27.84 wt% and enhances HHA selectivity in hydrochar by 13.57%, while shifting structures toward more oxidized and hydrophilic forms [60]. Iron oxides further catalyze condensation through Fe^3^^+^–catechol complexation and redox cycling between Fe^3^^+^ and Fe^2^^+^ [61]. Future work should clarify the roles of iron valence states and counter‐anions (e.g., Cl^−^, SO_4_^2^^−^), while considering potential risks such as soil acidification and toxicity from excessive iron inputs.

Layered double hydroxide (LDH) catalysts enhance hydrolysis and subsequent humification by providing tunable acid–base sites and stable layered structures. In sewage sludge and protein‐rich substrates, Ni–Mg–Al LDHs suppress Maillard reactions and reduce nitrogen fixation via acidic sites, while basic sites enhance protein hydrolysis and cell disruption. Elevated temperatures further accelerate these processes, promoting protein degradation and subsequent HHA formation (Figure 5b) [62]. The metal‐ion composition can be tuned to optimize functionality, although catalyst recovery and safety remain challenges.

Acids such as H_2_SO_4_ and H_3_PO_4_ solubilize biomass, accelerate hydrolysis, and promote the selective formation of HHA with condensed aromatic or mixed hydrophobic–hydrophilic structures. Feedstock composition strongly influences HHA properties. H_2_SO_4_ treatment yields hydrophobic, condensed HHA from sugarcane bagasse and distillers’ grains, whereas H_3_PO_4_ produces HHA with mixed hydrophilic–hydrophobic domains and more stable molecular architectures [40]. Although acid‐assisted HTH improves yield and stability, the corrosive conditions require neutralization and corrosion‐resistant operation.

### 4.2 Condensation‐Driven HTH Strategies

Alkaline conditions enhance lignin solubility through deprotonation of phenolic and carboxyl groups, promoting phenolic oxidation–condensation into HHA [63]. At pH 7–13, most HHA remains in the hydrochar (∼65%) with a smaller fraction in the aqueous phase (∼17%), whereas at pH >13, stronger deprotonation increases HHA dissolution into the liquid phase [64]. When combined with catalysts or oxidative aids, alkaline HTH can tailor HHA structure and functionality and enable the production of bio‐effective liquid fertilizers [65, 66] (Figure 3k).

Under alkaline conditions, Fe^3^^+^/Fe^2^^+^ catalysts accelerate biomass decomposition and influence HHA structure and bioactivity [33]. FeCl_3_ pretreatment lowers lignin decomposition temperatures and increases HHA yield while shortening reaction times [67]. For example, HHA yield from stalk residues increases from 6.77% to 14.08% and carbon content from 38% to 63.7%, while reducing retention time from 24 to 6 h [43]. Although less aromatic, the resulting products incorporate unstable carbon fragments derived from condensed intermediates, which form organic acids (e.g., lactic and hydroxysuccinic acid) and become embedded within the HHA matrix (Figure 5a). Iron oxides further promote oxidative hydrolysis, generating aromatic precursors that condense into bioactive molecules such as vanillin and methionine sulfoxide [33]. While these additives enhance functionality, they may reduce structural stability under extreme pH conditions.

Ca(OH)_2_ and CaO provide both OH^−^ and Ca^2^^+^ during HTH. OH^−^ promotes lignin depolymerization, while Ca^2^^+^ forms complexes with carboxyl groups, improving carbon retention and producing near‐neutral liquors with low phytotoxicity [37, 68]. Calcium additives also suppress excessive furan formation and facilitate the incorporation of lipid‐ and polysaccharide‐derived intermediates into HHA [34, 37].

Carbonates buffer alkalinity and promote HHA formation under relatively mild conditions, while offering low cost and environmental compatibility. In the co‐humification of sugarcane bagasse, leaves, and fruit peels, Na_2_CO_3_ addition increases HHA selectivity in hydrochar by 11.3% at 160°C, 6.2% at 180°C, and 5.1% at 220°C compared with controls [69]. Mn^2^^+^ additives accelerate hydrolysis and condensation via redox cycling and reduce HHA molecular weight, facilitating precursor aggregation into humified structures [44]. Ca‐based alkalis are inexpensive and environmentally compatible [37], whereas excessive dosing may cause salinization or pore blockage. Carbonates are similarly attractive, but their catalytic roles remain unclear, warranting further investigation and evaluation with complementary catalysts for selective HHA production.

### 4.3 Oxidation‐Controlled HTH Strategies

Hydrothermal oxidation introduces oxidants such as hydrogen peroxide, oxygen, and manganese‐based additives, which generate phenoxy radicals and reactive oxygen species, enabling the rapid formation of oxygen‐ and nitrogen‐rich HHA via polyphenol and Maillard reactions.

H_2_O_2_ produces •OH and •OOH radicals that oxidatively cleave biomass polymers at moderate temperatures (110°C–130°C) and repolymerize intermediates (e.g., furfural and 5‐HMF) into HHA enriched in carbonyl and carboxyl groups [70] (Figure 5b). H_2_O_2_‐assisted HTH is particularly effective for recalcitrant feedstocks, although it requires careful control to prevent rapid decomposition and safety risks.

O_2_‐assisted HTH significantly enhances depolymerization and condensation into HHA compared with N_2_ environments (Figure 3l). Under pressures up to 40 bar, proteins and lignocellulose are effectively degraded, producing HHA with higher aromaticity, hydrophobicity, and functional group density, indicative of enhanced chemical stability and environmental persistence [18]. Oxygen enables HHA yields of up to 31.3% at relatively mild temperatures, comparable to those obtained only at higher temperatures under inert conditions, while promoting the incorporation of carboxyl and nitrogenous‐containing groups [36]. Optimization of O_2_ dosage and residence time is essential to avoid over‐oxidation and increased operational costs.

MnO_2_ promotes biomass precursor degradation via Mn^3^^+^/Mn^2^^+^ redox cycling and generates •OH radicals that introduce carboxyl and hydroxyl groups into HHA [71]. Mn‐assisted systems further enhance Maillard and Mannich reactions of protein‐ and lignocellulose‐derived hydrolysates, yielding structurally complex HHA/HFA [72]. Manganese‐based oxidants thus provide a tunable route for producing oxygen‐rich humic products.

Although HTO involves oxidative drivers similar to wet oxidation, it differs in oxidation intensity, resulting in distinct transformation pathways and products. Wet oxidation favors extensive mineralization to low‐molecular‐weight compounds or CO_2_, rather than the formation of HHA.

### 4.4 Integrated and Tunable Process Designs

Pre‐hydrolysis increases biomass solubility and releases soluble intermediates and lignin‐rich fragments that enhance subsequent HHA formation. Two‐stage hydrothermal–enzymatic pretreatment increases HHA yield from vinegar residue to 15.3% [47], and acid hydrolysis similarly promotes depolymerization by generating glucose, xylose, and furfural, which more readily condense under alkaline HTH [58, 73] (Figure 5c). Lowering pH from 7 to 0 substantially increases HHA content (from 22.6 to 68.8 wt.%) [58], and recycling hydrolysate‐rich liquors provides further gains [59]. Although hydrolysis shortens reaction time and improves carbon conversion, the use of corrosive acids highlights the need for greener pretreatment methods and efficient liquor‐recycling strategies.

Oxidation‐assisted HTH couple oxidants with alkaline conditions by generating reactive oxygen species (e.g., O_2_^−^•, HOO•), which accelerate lignin depolymerization (via cleavage of β–O–4 and C–C linkages and oxidation of aliphatic carbon) while increasing carboxyl content and enhancing solubility and nutrient functionality (Figure 4d). Pressurized oxygen increases carboxyl content from 0.16 to 2.26 mmol g^−^^1^ in lignin‐derived HHA and from 3.2% to 12.6% in corn stalk‐derived HHA at 20 bar O_2_ [27, 74]. Advanced oxidation systems (e.g., persulfate‐KOH) further intensify Maillard chemistry and aromatic ring opening via •OH and SO_4_•^−^ radicals, enabling high carbon recovery and strong biostimulant properties [65].

Post‐oxidation of hydrochar or process liquors using HNO_3_, H_2_O_2_, or air heating increases HHA yield, aromaticity, and functional‐group density. Nitric acid treatments (e.g., 30% HNO_3_ at 70°C) enhance alkaline‐soluble HHA and introduce oxygenated functional groups while enlarging pore structure, outperforming thermal oxidation at 240°C [75]. Hydrogen peroxide post‐oxidation of alkaline HTH liquors increases HHA yield from 0.02 to 2.9 g L^−^^1^ and enriches carboxyl and hydroxyl functionalities, although excessive oxidation leads to structural degradation [43, 76] (Figure 5e). Air heating provides a low‐cost alternative, achieving >45% carbon conversion and HHA yields of up to 37% within 2 h [42]. The resulting HHA exhibits enhanced redox activity, metal binding, and nutrient‐delivery performance.

## 5 Soil Carbon Management and Environmental Performance

Soil degradation, including structure destruction, organic matter depletion, nutrient imbalance, and heavy metals accumulation, poses a major threat to long‐term agricultural sustainability [11, 77]. HHA, produced from diverse biomass feedstocks under tunable hydrothermal conditions, provides a multifunctional strategy for restoring soil quality, improving nutrient use efficiency, enhancing plant productivity, and mitigating contaminant risks (Figure 5a).

### 5.1 Soil Enhancement and Nutrient Cycling

**FIGURE 6:**
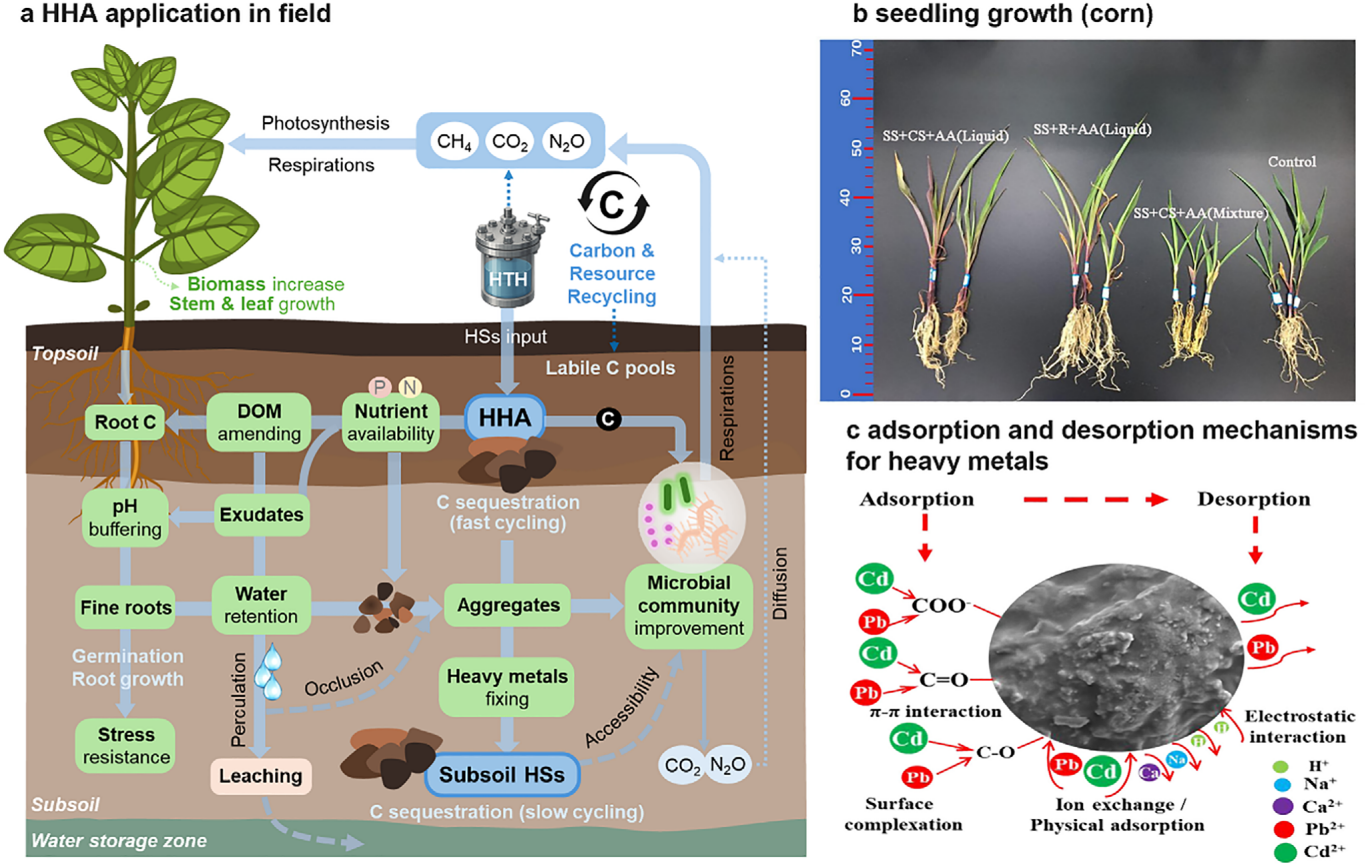
Field applications and contaminant interactions of HHA. (a) Major functions of HHA in agricultural soils, including nutrient retention and activation, soil structure improvement, and pollutant immobilization. (b) The application of HTH liquids increased soil available phosphorus (AP) by 1.43‐fold compared with controls, with AP levels gradually declining during cultivation in parallel with plant growth. Copyright 1983, Elsevier. Reproduced with permission [80]. (c) Summary of Pb(II) and Cd(II) adsorption–desorption mechanisms by HHA, highlighting the roles of electrostatic interaction, ion exchange, surface complexation, and π–cation interaction. Copyright 1983, Elsevier. Reproduced with permission [82].

HHA is enriched in oxygen‐containing functional groups and exhibits hybrid aromatic–aliphatic structures that enhance SOM content, water retention, and nutrient availability. The incorporation of HHA or HHA‐rich hydrochar consistently increases SOM and reduces carbon loss. For instance, the addition of 50% anthropogenic soil containing rice straw–derived hydrochar increases organic carbon in weak soil from 0.83% ± 0.18% to 7.21% ± 0.09% and SOM from 3.48% ± 0.22% to 11.53% ± 0.42% within 30 days [78]. Lincomycin fermentation residue–derived HTH liquid, containing 17.29 g L^−1^ HHA and 2.9 g L^−1^ amino acids, also significantly improves SOM [49]. Similarly, nitrogen‐rich HHA derived from corn‐straw hydrolysates and biogas slurry increases SOM by 6.9% relative to chemical fertilizer and by 4.3% compared with untreated soil [45]. Stabilization of organic matter arises from enhanced aggregation, cation exchange, and the formation of condensed aromatic structures that resist microbial decomposition [45] (Figure 6a).

HHA improves soil nutrient cycling through three mechanisms: (i) retention of cationic nutrients (NH_4_^+^, K^+^, Ca^2^^+^) via surface functional groups. In rice soils, HHA application, either alone (8 mL kg^−^^1^) or combined with a 30% reduction in chemical nitrogen fertilizer (0.2–0.4 mL kg^−^^1^), increases nitrate nitrogen (up to 0.84–6.90‐fold depending on rhizosphere zone) and reduces electrical conductivity by 28.77%–100.2% relative to conventional fertilization [79]. Co‐application with urea (200 mg N kg^−^^1^ soil; urea:HHA = 3:1) further reduces nitrogen loss by 40%–50%, although highly aromatic HHA may partially inhibit biological N fixation [55]; (ii) activation of mineral‐bound phosphorus. In sludge‐derived systems, dissolved phosphorus increases from 7045 to 10075 mg L^−^^1^ under optimized conditions [80]. In black and saline–alkali soils, increasing HHA dosage (0–600 mg kg^−^^1^) reduces P breakthrough peaks (C/C_0_) from 0.86 to 0.69 and 0.89 to 0.71, respectively [81]; (iii) stimulation of microbial N fixation. HHA amendment (10%–50%) in low‐fertility soil promoted microbial community reconstitution, enhancing nitrogen cycling through stimulation of N‐fixing bacteria such as *Herbaspirillum frisingense*. Ammonium nitrogen increases from 8.73 ± 0.25 mg kg^−^^1^ (CK) to 9.80 ± 0.60, 12.32 ± 0.32, and 14.32 ± 0.75 mg kg^−^^1^, respectively [78].

Soil physical properties, including aggregation, porosity, aeration, hygroscopicity, and swelling, are also significantly influenced by HHA [49]. The application of 50% HHA improves the water‐holding capacity of weak soil from 53.7% to 80.0%, primarily due to decreased bulk density (from 1.05 to 0.80 g cm^−^^3^) and increased porosity of the HHA–soil mixture (11.65%) compared to the soil alone (4.87%) [78]. These structural improvements enhance infiltration, aeration, and soil–plant–water interactions. HHA typically exhibits a near‐neutral pH (6.5–7.5) and high cation‐exchange capacity [34], enabling it to buffer acidic and saline–alkaline soils and moderate pH fluctuations caused by fertilizer inputs [45]. Although synthetic HHA–urea complexes may cause a transient decrease in pH [55], subsequent buffering by HHA stabilizes soil pH and minimizes fluctuations, thereby promoting chemically resilient soil environments.

### 5.2 Plant Performance and Stress Resilience

HHA exerts broad plant growth–promotion effects by enhancing nutrient uptake, stimulating root and shoot development, and improving photosynthetic and metabolic performance (Figure 6b). During HTH, nitrogen is largely converted into plant‐available NH_4_^+^, while P and K concentrations increase, enabling HHA to function as an efficient nutrient carrier [23]. Chelation and redox‐active functional groups further enhance micronutrients uptake, particularly for Mn and Fe [44, 83]. In addition, phosphorus availability increases markedly, with soluble and available P reaching 3.75‐ and 6.65‐fold higher levels than the control [80].

HHA functions as a biostimulant that promotes seed germination and early seedling vigor by activating cellular metabolism, cell‐division pathways, and auxin‐like signaling. Rice straw‐derived HHA increases rice germination from 56.9% to 81.8% [78], and performs comparably to natural HA, largely owing to its abundant phenolic and indole‐like constituents [17, 33]. However, over‐condensed N‐heterocycles (e.g., melanoidins) suppressed carbohydrate metabolism and inhibit germination, underscoring the need to avoid over‐aromatization during HTH [25, 82].

Through auxin‐like activity, enhanced nutrient supply, and metabolic regulation, HHA derived from rice straw [78], lignin [27], sludge [79], bagasse [40], lincomycin‐residue [49], and lichi‐wood [37] effectively promotes shoot elongation and biomass accumulation, with performance comparable to that of mineral fertilizers [45, 49, 59, 80]. For example, lignin‐derived HHA increases maize height by 12%–27% and dry weight by up to 92% [27], while more amphiphilic HHA releases bioactive compounds more readily and supports stronger root development than hydrophobic variants [40]. These effects involve coordinated physiological responses. Sludge‐derived HHA modulates indole‐3‐acetic acid signaling and plasma membrane H^+^‐ATPase activity [25], whereas Fe‐catalyzed HHA upregulated *OsRHL1* and *OsZPT5‐07*, increasing root activity by 167% and photosynthetic rate by 72% [33]. HHA also activates the tricarboxylic acid cycle, elevating soluble sugar and protein levels [25].

HHA enhances photosynthetic efficiency by promoting chloroplast development and improving coordination between light reactions and the Calvin–Benson cycle [38, 49]. Total chlorophyll in rice seedlings increases from 9.51 to 12.59 mg L^−^^1^ with the addition of 10% HHA‐rich anthropogenic soil to low‐fertility soil [78], while chlorophyll content in maize increases by 14%–32% following the application of oxidized HHA [27]. HHA also enhances PSII quantum efficiency, electron transport, and quinone reoxidation kinetics [84], while modulating stomatal conductance, reducing intercellular CO_2_, and improving ATP utilization [84]. Feedstock origin shapes HHA performance, with cyanobacterial‐derived HHA enhancing photochemical conversion and sludge‐ and lignocellulose‐derived HHA supporting photosynthetic enzymes through cofactor and nutrient supply [84]. Upregulation of chloroplast‐related genes such as *OsDCL* further increases photosynthetic rates (by 72%) and transpiration [33].

Plants exhibit improved tolerance to drought and salinity following HHA treatment through enhanced ionic homeostasis, redox balance, and antioxidant activity [49]. Sugarcane bagasse‐derived HHA reduces malondialdehyde levels in salt‐stressed maize by 35% and increases superoxide dismutase and peroxidase activities [17], while rice straw‐derived HHA (pH 4.85) enhances K^+^ uptake, restores ionic balance, and reduces bacterial wilt in rice by up to 50% [78]. Upregulation of stress‐responsive genes (e.g., *DREB* and *LEA*) and activation of phenolic‐flavonoid pathways further increase soluble sugar and protein contents, as well as stress‐related metabolites such as *p*‐coumaric acid [33].

HHA generally exhibits low phytotoxicity and maintains crop metal concentrations within regulatory limits. Its strong chelation capacity can keep toxic metal levels in grains (e.g., Hg, As, Pb, Cr, and Cd) within safety thresholds and reduce the bioavailability of metals such as Cu, Zn, and Pb [85], although moderate increases in Zn (4.5%–17.8%) have been reported [79]. HHA derived from vegetable waste [34] and lincomycin‐residue [49], characterized by higher aliphaticity and lower aromaticity, exhibits superior safety [19]. Overextended HTH durations (>12 h) or contaminated feedstocks may lead to the accumulation of toxic compounds [25]. Therefore, careful feedstock selection and process control are essential to prevent the accumulation of toxic fractions and ensure product safety [12].

### 5.3 Ecological Functions and Environmental Safety

HHA plays a dual role in soil ecological regulation and environmental remediation by immobilizing contaminants and reshaping soil microbial communities. Its densely oxygenated functional groups and hybrid aromatic–aliphatic structures enable strong complexation with metal cations and interaction with organic pollutants, thereby reducing their mobility and bioavailability [86] (Figure 6c). Compared with natural HA, HHA typically contains higher oxygen functionality, resulting in stronger binding affinity; its Cu‐complexation capacity is reported to be fourfold greater than that of Amazonian soil‐derived HA [87]. Oxidation further enhances reactivity, as oxidized HHA with increased carboxyl density and surface area shows nearly threefold higher Cd^2^^+^ removal through strengthened chemisorption [74]. Nanomaterial–HHA hybrids further amplify these benefits. For example, magnetic biochar–HHA composites can adsorb up to 169.7 mg g^−^^1^ of Cd^2^^+^ (10 mg adsorbent in 40 mL of a 50 mg L^−^^1^ solution) [88], while HHA‐stabilized nano‐zero‐valent iron achieves adsorption capacities exceeding 649 mg g^−^^1^ of Pb^2^^+^ (10 mg in 40 mL of an 80 mg L^−^^1^ solution), owing to suppressed nanoparticle aggregation and enhanced reductive activity [89]. Therefore, HHA serves as an effective multifunctional agent for metal immobilization, attenuation of persistent organic pollutants, and pathogen suppression.

Beyond contaminant remediation, HHA substantially enhances soil microecology. Acting as a bioactive carbon source and redox mediator, it enriches beneficial microbial guilds involved in nitrogen fixation, phosphorus solubilization, and carbon cycling, while suppressing phytopathogenic bacteria and fungi. In reconstructed black soils, HHA restores microbial community structure within 30 days, elevating Proteobacteria abundance to 77%–87% [78] and decreasing pathogenic Bacillaceae from 5.8% to 0.4% [45]. Fungal communities shift toward beneficial taxa dominated by Ascomycota and fertility‐enhancing Humicola [78]. Enhanced populations of nitrogen‐fixing microorganisms (e.g., Proteobacteria, Actinobacteria, endophytic OTU130) [55, 78], and phosphorus‐solubilizing bacteria (e.g., *Pseudomonas*) [45] improved nutrient utilization efficiency. Beneficial plant–microbe interactions are strengthened, with *Glutamicibacter* increasing two‐ to threefold and pathogens such as *Fusarium* and *Chaetomium* declining by 40%–60% [45]. The antimicrobial effects of HHA vary with feedstock composition. Sulfur‐rich, aliphatic sweet potato‐derived HHA strongly inhibits *Botrytis cinerea* and *Phytophthora* capsici [19], while HTH liquors from lincomycin residues suppress pathogens by 30–50% through volatile organic acids without inducing phytotoxicity [49].

In addition, although some HTH by‐products, such as carbohydrate‐derived non‐aromatic species and phenolics, can support soil functioning as microbial substrates, the process may also generate potentially phytotoxic intermediates, including melanoidins, organic acids, and furan derivatives (e.g., HMF and furfural) [24, 90, 91, 92]. Given that many inhibitory compounds are water‐soluble, solid‐phase HHA is generally considered safer for soil application, particularly when hydrochar contains low levels of dissolved organic matter. However, excessive reaction severity may lead to over‐aromatization and condensation, producing highly condensed structures with reduced functional group availability, reactivity, and bioavailability, thereby creating a trade‐off between carbon stability and biological activity. In addition, contaminated feedstocks (e.g., sewage sludge or manure) may introduce heavy metals into HTH‐derived products, underscoring the importance of feedstock selection and quality control.

Ecotoxicological assessment typically integrates bioassays and biochemical indicators (e.g., seed germination index, root elongation, soil respiration, and microbial activity), and phytotoxicity is strongly concentration‐dependent under realistic application scenarios. These risks can be effectively mitigated through process optimization and post‐treatment strategies, such as extraction, dilution, or stabilization of phenolic‐ and furan‐rich fractions [92, 93], as well as adjustment of reaction conditions (e.g., alkaline media or CaO addition) [39, 66, 94]. Future work should further elucidate the structure–function relationships governing HHA interactions with emerging contaminants (e.g., PFAS, antibiotics, and petroleum hydrocarbons), as well as the microbial pathways influenced by different HHA chemotypes, and establish standardized toxicity thresholds and classification frameworks that account for feedstock variability and regulatory requirements.

### 5.4 Carbon Permanence and Stabilization

The incorporation of HHA into soils enhances long‐term carbon persistence through a combination of intrinsic material properties and soil–microbe–mineral interactions [55]. HHA is enriched in condensed aromatic structures and oxygenated functional groups, such as quinones, carbonyls, and carboxyls, which impart high chemical stability and resistance to microbial degradation. These features substantially suppress carbon mineralization, reducing cumulative CO_2_ emissions by 30–45% when straw‐derived HHA is applied at low dosages (50–100 mg kg^−^^1^) [95].

HHA also reshapes the soil biochemical environment in ways that favor carbon retention. Its functional groups dampen the activity of carbon‐degrading enzymes and reorganize microbial communities toward carbon‐conserving guilds, including increased abundance of carbon‐fixing taxa such as Chloroflexi and *Rubrivivax* gelatinosus [95]. Associated shifts in dissolved organic carbon dynamics constrain microbial decomposition and reinforce stable soil organic carbon pools. In parallel, HHA improves soil aggregation, porosity, and moisture retention, limiting microbial accessibility to carbon substrates. Strong interactions with minerals, soil water networks, and native SOM promote the formation of persistent mineral–organic complexes, reducing SOM leaching by up to 16.9% and mitigating SOM decline during cultivation [45].

At the systems scale, HHA provides sustained SOM replenishment and slows the rapid carbon loss typical of degraded or intensively fertilized soils. Agricultural residues can yield 300–500 kg of HHA per tonne of biomass, with a carbon sequestration efficiency of 40%–60%, underscoring the potential of HTH as a negative‐emissions pathway [84]. Nonetheless, carbon permanence remains sensitive to feedstock composition, degree of aromatic condensation, oxidation state, and soil type. Future work should elucidate multiscale interactions among HHA, minerals, soil water, native SOM, and microbial consortia to enable predictive models of carbon stabilization across temporal and environmental gradients.

## 6 Sustainability Assessment of HTH Technology

### 6.1 Life‐Cycle Assessment

**FIGURE 7:**
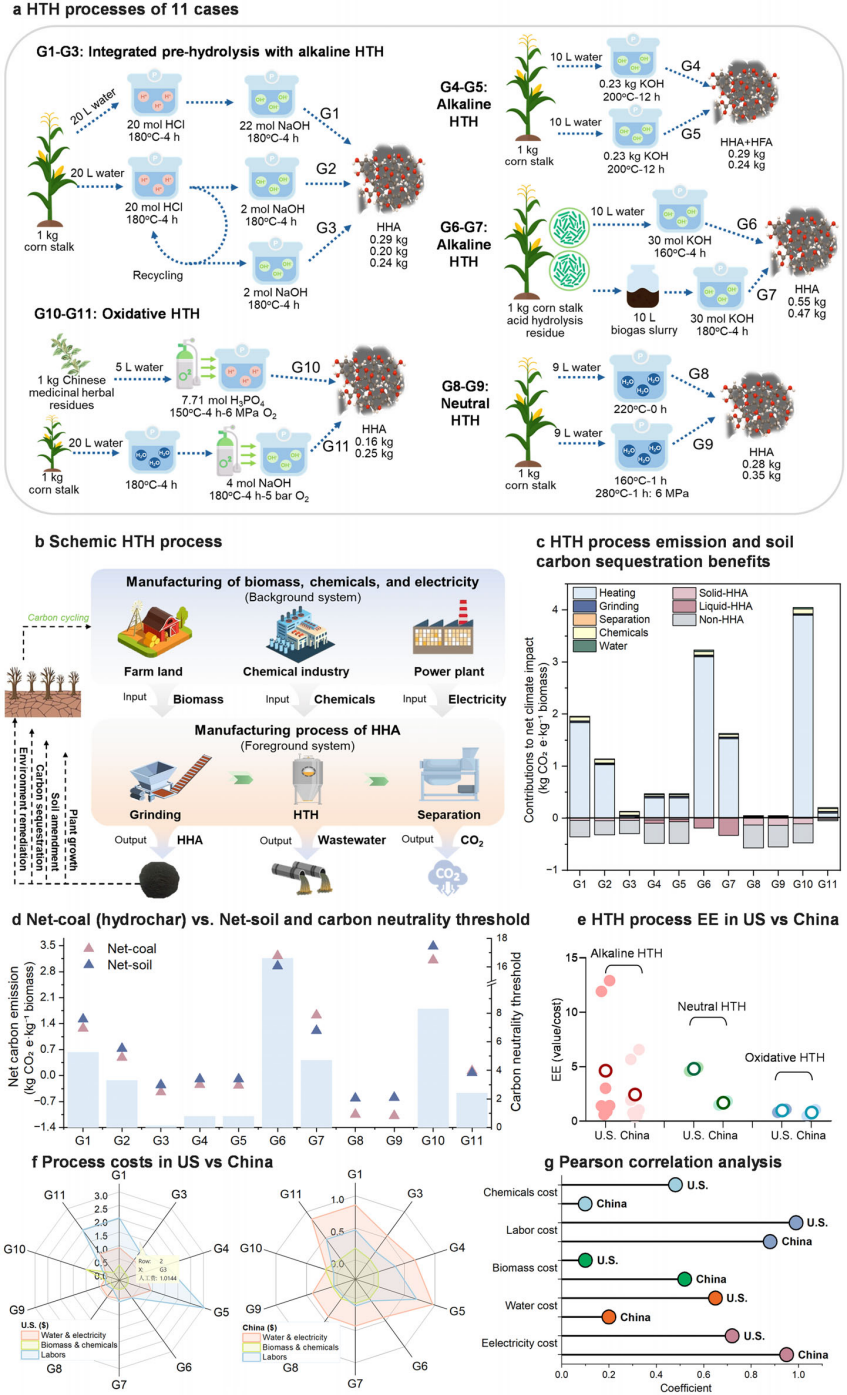
Environmental and technoeconomic assessment of HTH. (a), Schematic of HTH showing major material and energy inputs and product outputs. (b), Simplified process flow of HTH highlighting grinding, heating, and solid–liquid separation steps. (c), Process greenhouse‐gas emissions and soil‐carbon sequestration benefits, expressed as positive values for emissions and negative values for carbon storage. (d), Comparison of fuel‐substitution credits (Net‐coal) and soil‐carbon sequestration benefits (Net‐soil), including carbon‐neutrality thresholds for each scenario. (e), EE calculated as the ratio of total cost to HHA market value. (f), Cost structure of HTH processes in the U.S. and China. U.S. reagent and utility prices (USD kg^−^^1^ unless noted): NaOH 0.0237, HCl 0.275, H_3_PO_4_ 1.12, corn stalk 0.05, water 7.73 USD t^−^^1^, electricity 0.12 USD kWh^−^^1^, labor 35.5 USD h^−^^1^ (three‐shift schedule). Product values: HA 10 USD kg^−^^1^ (G1–G3, G8–G11) and potassium‐based HA 24 USD kg^−^^1^ (G4–G7). In China: NaOH 0.12, HCl 0.01, H_3_PO_4_ 0.94, corn stalk 0.07, herbal residues 0.14, water 0.68 USD t^−^^1^, electricity 0.09 USD kWh^−^^1^, labor 8.19 USD h^−^^1^; product values: HA 2.8 USD kg^−^^1^ and potassium‐based HA 7.5 USD kg^−^^1^ [101, 102, 103, 104, 105, 106, 107, 108]. (g), Pearson correlation analysis highlighting heating demand and labor cost as major economic drivers.

The environmental performance of HTH is evaluated by quantifying (i) CO_2_ emissions from major unit operations and (ii) soil carbon sequestration from HHA‐enriched hydrochar and dissolved HHA. Eleven representative cases are assessed (Figure 7a) across alkaline (G1–G7) [28, 59], neutral (G8–G9) [32, 50], and oxidative (G10–G11) [36, 74] routes. Under an industrial boundary that includes electricity for heating, grinding, mixing, and separation, as well as embodied emissions from chemicals, process emissions ranged from 0.05 to 4.05 kg CO_2_e per kg biomass, with thermal demand as the dominant contributor (Figure 7b,c; Table S1). Reagent use, particularly NaOH and KOH, also contributes to upstream emissions and wastewater burdens. Neutral routes (G8–G9) exhibit the lowest intensities (0.049–0.051 kg CO_2_e kg^−^^1^) due to minimal reagent use and moderate reaction severity, whereas partial substitution with Ca(OH)_2_ and high water recycling (90%–95%) can further reduce emission by approximately 0.8–1.2 t CO_2_ per ton of straw processed [37].

HTH also converts biomass carbon into soil carbon pools. Hydrochar yields (6.8%–58%) with 45%–60% C content correspond to an estimated carbon sequestration potential of 0.36–0.75 kg CO_2_e kg^−^^1^ biomass, driven by enriched HHA (7%–55%) and aromatically condensed structures (Tables S2 and S3). Dissolved HHA (6.8–46.8% yield; 54–76% C) contributes an additional 0.06–0.28 kg CO_2_e kg^−^^1^ through organo–mineral complex formation (Figure 7c; Table S4). However, it is important to distinguish between operational stability and long‐term carbon permanence. Tracking the long‐term transformation of HHA in soils remains challenging, as exogenous HHA becomes mixed with native humic substances (HA and FA), making its aging and stabilization processes difficult to distinguish. In this work, stabilization factors (α_solid_: 0.5–0.9; α_liquid_: 0.3) are used as scenario‐based parameters informed by hydrochar stability [96, 97, 98] and supported by biochar aging literature [99, 100]. These estimates, although reasonable as scenario‐based approximations, remain uncertain and do not represent centennial‐scale persistence, particularly for dissolved HHA, which is influenced by organo–mineral interactions, microbial turnover, and leaching losses. Under these assumptions, G3–G5 and G8–G9 approach or achieve net emissions reductions (Figure 7d; Table S4). Only neutral (G8–G9) and moderate‐alkaline routes (G3–G5) exhibit potential for net‐negative GHG balances, whereas high‐alkali and oxidative pathways remain net‐positive. Carbon‐neutrality thresholds further indicate that these latter pathways require substantially higher carbon stabilization to reach neutrality (Table S5). Sensitivity analysis confirms that net emissions decrease with increasing solid‐phase stabilization, while the relative ranking of scenarios remains largely unchanged (Table S6).

These results indicate that HHA‐enriched hydrochar is a key contributor to potential carbon retention, whereas dissolved HHA alone is insufficient to offset process emissions, although it may also contribute to plant growth promotion. Stabilizing HHA within the hydrochar matrix, where it forms mineral‐associated and aromatically condensed domains, may enhance persistence. However, its long‐term effectiveness still requires validation under field conditions. Importantly, the return of HTH‐derived products to soil may also stimulate the transformation and re‐association of low‐molecular‐weight organic matter in soil, thereby contributing to increased SOM content [78]. This effect not only enhances SOM levels but may also support longer‐term carbon stabilization through organo–mineral interactions and secondary humification processes.

Future optimization should therefore prioritize maximizing solid‐phase carbon retention, enhancing HHA incorporation and condensation within hydrochar, and steering carbon toward more recalcitrant fractions. When coupled with strategies such as reagent substitution, heat and water recovery, and process intensification, HHA‐enriched hydrochar may provide a promising pathway toward low‐emission or potentially carbon‐neutral to carbon‐negative HTH systems.

### 6.2 Technoeconomic Analysis

The techno‐economic performance of HTH systems is evaluated by quantifying (i) the cost of processing each kilogram of biomass to produce humic‐rich hydrothermal products, and (ii) the relationship between production cost and market value to assess commercial feasibility. Eleven representative process pathways are examined under U.S. and China cost structures. The cost model integrates both capital and operating expenditures, including feedstock, chemical reagents, electricity, water consumption, labor, and downstream product valuation. Economic performance was strongly governed by product market value. As shown in Figure 7e, higher selling prices for HHA‐based soil amendments in the U.S. lead to greater economic efficiency (EE), with potassium‐enriched products providing the highest economic return due to their higher market premiums.

Operational costs vary markedly between regions. Total production costs were consistently higher in the U.S., primarily driven by elevated electricity, labor, and water prices, despite comparatively lower feedstock and chemical costs (Figure 7f). Notably, KOH used in the HTH process can partially substitute conventional fertilizer inputs, thereby offsetting part of the chemical cost rather than representing a net loss. Under U.S. conditions, G5 exhibits the highest unit cost due to its extended residence time (24 h), whereas the neutral pathway G8 achieves the lowest cost (0.61 USD kg^−^^1^) owing to shorter reaction duration and minimal reagent demand. In China, electricity consumption dominates operating expenses, while labor costs were significantly lower. Pearson correlation analysis (Figure 7g) further indicates that these differences arise from the distinct cost structures in the two regions. In the U.S., higher labor expenses amplify the contribution of labor to total cost, whereas in China, relatively lower labor costs shift the economic burden toward energy consumption.

The techno‐economic viability of HTH systems depends on both process intensification and the realization of value from HHA‐rich products and remains sensitive to market fluctuations. If HHA market prices fall below 4 USD kg^−^^1^ in the U.S. or 2 USD kg^−^^1^ in China, HTH processes become economically marginal unless improvements in yield, co‐product valorization, or by‐product credits are achieved. It is therefore important to emphasize region‐specific optimization strategies, such as reducing labor intensity in the U.S. and improving energy efficiency in China.

In addition, economic feasibility is strongly influenced by process scale, system boundaries, and integration with agricultural practices. Strategies such as optimizing reaction severity, enhancing process automation, and targeting higher‐value soil amendment markets are essential for scale‐up. These approaches can reduce unit costs through improved energy efficiency, reduced labor demand, and lower capital intensity per unit output, thereby supporting the scalable deployment of HTH systems.

## 7 Summary, Challenges, and Future Perspectives

HTH has emerged as a promising clean technology for converting biomass waste into HHA to improve soil fertility, remediate pollutants, restore soil microenvironments, and promote plant growth. The HTH pathway offers several advantages for HHA production, including tunable product properties, short reaction times, and relatively high carbon efficiency. A unified mechanistic understanding that links reaction pathways, molecular evolution, and structure–function relationships across hydrolysis‐, condensation‐, and oxidation‐dominated regimes is essential for the development and deployment of efficient HTH technologies. Despite growing interest in this technology, several critical issues remain insufficiently understood and continue to limit wider application. Key challenges include the long‐term impacts of HHA on soil carbon sequestration, overall HTH efficiency, the mitigation of HHA‐related phytotoxicity, as well as the need for stronger policy support and commercial‐scale deployment.

### 7.1 Long‐Term Carbon Sequestration

Current evidence demonstrates the short‐term benefits of HHA for soil carbon stabilization, yet their long‐term impacts on soil–atmosphere carbon cycling remain poorly constrained. Future studies should integrate process‐based models, big‐data analytics, and long‐term field monitoring to evaluate carbon permanence and system‐level responses. In addition, the development of standardized carbon accounting methodologies and carbon‐credit frameworks will be essential to quantify the climate benefits of HTH‐derived products and support their integration into carbon markets. These advances will be critical for establishing HTH as a credible clean‐technology pathway for CO_2_ mitigation and durable soil carbon sequestration.

### 7.2 Efficiency and De‐Phytotoxicity

As a complex thermochemical process involving hydrolysis, condensation, and decarboxylation, HTH requires more systematic kinetic studies supported by advanced analytical techniques, particularly operando and in situ spectroscopic methods, to resolve the dynamic transformations of intermediates. Coupling these approaches with data‐driven and AI‐assisted modeling may further accelerate mechanistic understanding and process optimization. In parallel, managing phytotoxicity remains critical for practical applications. Although HTH products may contain transient inhibitory intermediates, their risks can be effectively mitigated through process optimization, post‐treatment stabilization, and controlled application strategies. Furthermore, while specific standards for HHA are still evolving, compliance with existing regulations for organic soil amendments remains essential to ensure environmental safety.

### 7.3 Policy Support and Commercial Scale‐Up

Standardized HHA quality classifications and soil carbon measurement protocols remain inadequate, hindering broader deployment and carbon‐credit verification. Scaling up HTH also requires the evaluation of integrated systems, including coupling with bioenergy production in BECCS‐like configurations (bioenergy with carbon capture and storage) and existing wastewater treatment infrastructures, to enhance carbon stabilization while reducing operational costs. Stronger policy frameworks, certification schemes, and economic incentives will further accelerate commercial adoption. In addition, dedicated standards and regulatory frameworks for HHA should be further developed to ensure environmental and biosafety.

## Author Contributions

Z.L. and Z.Y. contributed to the conceptualization. Z.L. wrote the article, researched data, and visualized the figures. Z.Y., H.Z., and L.Z. led the overall conceptual design and activity. All authors reviewed and/or edited the manuscript before submission.

## Conflicts of Interest

The authors declare no conflicts of interest.

## Supporting information




**Supporting File**: advs75558‐sup‐0001‐SuppMat.docx.

## Data Availability

The data that support the findings of this study are available from the corresponding author upon reasonable request.

## References

[1] C. Andrade Díaz , A. Albers , E. Zamora‐Ledezma , and L. Hamelin , “The Interplay Between Bioeconomy and the Maintenance of Long‐term Soil Organic Carbon Stock in Agricultural Soils: A Systematic Review,” Renewable and Sustainable Energy Reviews 189 (2024): 113890, 10.1016/j.rser.2023.113890.

[2] J. Weber , Y. Chen , E. Jamroz , and T. Miano , “Preface: Humic Substances in the Environment,” Journal of Soils and Sediments 18 (2018): 2665–2667, 10.1007/s11368-018-2052-x.

[3] E. Efremenko , N. Stepanov , O. Senko , I. Lyagin , O. Maslova , and A. Aslanli , “Artificial Humic Substances as Biomimetics of Natural Analogues: Production, Characteristics and Preferences Regarding Their Use,” Biomimetics 8 (2023): 613, 10.3390/biomimetics8080613. 38132553 PMC10742262

[4] H. Chu , S. Li , K. Sun , Y. Si , and Y. Gao , “Exolaccase‐Boosted Humification for Agricultural Applications,” iScience 25 (2022): 104885. 36039291 10.1016/j.isci.2022.104885 PMC9418857

[5] Y. Ma , D. Woolf , M. Fan , L. Qiao , R. Li , and J. Lehmann , “Global Crop Production Increase by Soil Organic Carbon,” Nature Geoscience 16 (2023): 1159–1165, 10.1038/s41561-023-01302-3.

[6] G. C. Dargie , S. L. Lewis , I. T. Lawson , et al., “Age, Extent and Carbon Storage of the central Congo Basin Peatland Complex,” Nature 542 (2017): 86–90, 10.1038/nature21048. 28077869

[7] A. G. Zavarzina , E. G. Kravchenko , A. I. Konstantinov , I. V. Perminova , S. N. Chukov , and V. V. Demin , “Comparison of the Properties of Humic Acids Extracted From Soils by Alkali in the Presence and Absence of Oxygen,” Eurasian Soil Science 52 (2019): 880–891, 10.1134/S1064229319080167.

[8] M. S. Diallo , A. Simpson , P. Gassman , et al., “3‐D Structural Modeling of Humic Acids Through Experimental Characterization, Computer Assisted Structure Elucidation and Atomistic Simulations. 1. Chelsea Soil Humic Acid,” Environmental Science & Technology 37 (2003): 1783–1793, 10.1021/es0259638. 12775049

[9] Y. Zhang , D. Yue , X. Wang , and W. Song , “Mechanism of Oxidation and Catalysis of Organic Matter Abiotic Humification in the Presence of MnO_2_ ,” Journal of Environmental Sciences 77 (2019): 167–173, 10.1016/j.jes.2018.07.002. 30573080

[10] R. Fründ , H.‐D. Lüdemann , F. J. Gonzalez‐Vila , G. Almendros , J. C. del Rio , and F. Martin , “Structural Differences Between Humic Fractions From Different Soil Types as Determined by FT‐IR and 13 C‐NMR Studies,” Science of the Total Environment 81 (1989): 187–194.

[11] F. Yang , N. Tarakina , and M. Antonietti , “A Stunt of Sustainability: Artificial Humic Substances Can Generate and Stabilize Single Fe 0 Species on Mineral Surfaces,” Chemsuschem 16 (2023): 202300385, 10.1002/cssc.202300385. 37010131

[12] Z. Li , Y. Shao , W. He , et al., “Insight Into the co‐hydrothermal Humification of Corn Stalk and Sewage Sludge for Enhanced Nitrogen‐rich Humic Acid Production,” Frontiers of Environmental Science & Engineering 18 (2024): 153, 10.1007/s11783-024-1913-3.

[13] S. A. Waksman , Humus: Origin, Chemical Composition, and Importance in Nature. (The Williams & Wilkins Company, 1936).

[14] Y. Zhan , R. Li , W. Chen , et al., “Deciphering the Carbon and Nitrogen Component Conversion in Humification Process Mediated by Distinct Microbial Mechanisms in Composting From Different Domestic Organic Wastes,” Environmental Science and Pollution Research 32 (2025): 13474–13486, 10.1007/s11356-024-32224-1. 38289551

[15] W. Chen , Y. Yang , S. Chang , et al., “Microbial Necromass Analogues Reshape Composting Humification Pathways,” Bioresource Technology 441 (2025): 133583, 10.1016/j.biortech.2025.133583. 41192488

[16] Y. Deng , H. Mao , X. Wang , Z. Zhao , and Y. Zhang , “Enhancing Humification and Digestate Maturity in High‐Solids Anaerobic Digestion of Agricultural Wastes Using Biochar,” Water Research 287 (2025): 124378. 40811985 10.1016/j.watres.2025.124378

[17] L. R. Bento , C. A. Melo , O. P. Ferreira , et al., “Humic Extracts of Hydrochar and Amazonian Dark Earth: Molecular Characteristics and Effects on Maize Seed Germination,” Science of The Total Environment 708 (2020): 135000, 10.1016/j.scitotenv.2019.135000. 31791776

[18] L. Pola , M. Movila , J. Erro , et al., “Structure of the Humic Acid‐Like Compounds of Raw and Hydrothermally Treated Sewage Sludge,” International Journal of Biological Macromolecules 242 (2023): 125115, 10.1016/j.ijbiomac.2023.125115. 37257533

[19] Y. Cao , H. Jin , N. Zhu , and Z. Zhou , “High‐efficiency Fungistatic Activity of Vegetable Waste‐based Humic Acid Related to the Element Composition and Functional Group Structure,” Process Safety and Environmental Protection 169 (2023): 697–705, 10.1016/j.psep.2022.11.028.

[20] E. M. Pena‐MÈndez , J. Havel , and J. Patočka , “Humic Substances—Compounds of Still Unknown Structure: Applications in Agriculture, Industry, Environment, and Biomedicine,” Journal of Applied Biomedicine 3 (2005): 13–24, 10.32725/jab.2005.002.

[21] R. T. Oglesby , R. F. Christman , and C. H. Driver , “The Biotransformation of Lignin to Humus Facts and Postulates,” Advances in Applied Microbiology 9 (1967): 171–184. 4866748

[22] S. Dou , J. Shan , X. Song , et al., “Are Humic Substances Soil Microbial Residues or Unique Synthesized Compounds? A Perspective on Their Distinctiveness,” Pedosphere 30 (2020): 159–167, 10.1016/S1002-0160(20)60001-7.

[23] N. Marzban , J. A. Libra , V. S. Rotter , et al., “Maximizing the Value of Liquid Products and Minimizing Carbon Loss in Hydrothermal Processing of Biomass: An Evolution From Carbonization to Humification,” Biochar 6 (2024): 44, 10.1007/s42773-024-00334-1.

[24] J. Chen , T. Sun , P. Yang , et al., “Inhibitory Effect of Microplastics Derived Organic Matters on Humification Reaction of Organics in Sewage Sludge Under Alkali‐Hydrothermal Treatment,” Water Research 252 (2024): 121231. 38324988 10.1016/j.watres.2024.121231

[25] S. Cai , Y. Zhang , A. Hu , et al., “Dissolved Organic Matter Transformation Mechanisms and Process Optimization of Wastewater Sludge Hydrothermal Humification Treatment for Producing Plant Biostimulants,” Water Research 235 (2023): 119910. 37001233 10.1016/j.watres.2023.119910

[26] S. Han , B. Zhang , M. Wang , R. Guo , and S. Fu , “Optimization of Alkaline Hydrothermal Treatment for Humic Acids Production From Corn Straw Digestate Using the Response Surface Methodology,” Journal of Environmental Chemical Engineering 12 (2024): 112465, 10.1016/j.jece.2024.112465.

[27] S. Sutradhar , N. Alam , L. P. Christopher , and P. Fatehi , “KOH Catalyzed Oxidation of Kraft Lignin to Produce Green Fertilizer,” Catalysis Today 404 (2022): 49–62, 10.1016/j.cattod.2022.08.007.

[28] X. Peng , S. Gai , K. Cheng , and F. Yang , “Hydrothermal Humification Mechanism of Typical Agricultural Waste Biomass: A Case Study of Corn Straw,” Green Chemistry 25 (2023): 1503–1512.

[29] R. Wang , X. Zheng , Z. Feng , et al., “Hydrothermal Carbonization of Chinese Medicine Residues: Formation of Humic Acids and Combustion Performance of Extracted Hydrochar,” Science of The Total Environment 925 (2024): 171792, 10.1016/j.scitotenv.2024.171792. 38508251

[30] M. Klavins , L. Ansone‐Bertina , J. Krumins , O. Purmalis , L. Klavina , and Z. Vincevica‐Gaile , “Hydrothermal Carbonization of Invasive Plant Biomass as a Tool for Its Safe Utilization and Production of Artificial Humic Substances,” Invasive Plant Science and Management 17 (2024): 86–94.

[31] P. Chen , R. Yang , Y. Pei , et al., “Hydrothermal Synthesis of Similar Mineral‐Sourced Humic Acid from Food Waste and the Role Of Protein,” Science of The Total Environment 828 (2022): 154440. 35276141 10.1016/j.scitotenv.2022.154440

[32] Z. Liu , J. Su , Z. Yao , Y. Zhang , L. Wang , and L. Zhao , “Enhancing Humic Acids Production From Cornstalk Under Fast Hydrothermal Conditions: Insights Into New Pathways of Skeleton Self‐polymerization and Branch Growth,” Bioresource Technology 406 (2024): 131020, 10.1016/j.biortech.2024.131020. 38909871

[33] Y. Zhi , X. Li , F. Lian , et al., “Nanoscale Iron Trioxide Catalyzes the Synthesis of Auxins Analogs in Artificial Humic Acids to Enhance Rice Growth,” Science of The Total Environment 848 (2022): 157536, 10.1016/j.scitotenv.2022.157536. 35878859

[34] M. Ghaslani , R. Rezaee , O. Aboubakri , et al., “Lime‐assisted Hydrothermal Humification and Carbonization of Sugar Beet Pulp: Unveiling the Yield, Quality, and Phytotoxicity of Products,” Biofuel Research Journal 11 (2024): 2025–2039, 10.18331/BRJ2024.11.1.4.

[35] L. Wang , Y. Chi , K. Du , Z. Zhou , F. Wang , and Q. Huang , “Hydrothermal Treatment of Food Waste for Bio‐fertilizer Production: Formation and Regulation of Humus Substances in Hydrochar,” Science of The Total Environment 838 (2022): 155900, 10.1016/j.scitotenv.2022.155900. 35588799

[36] M. Xu , P. Chen , J. Wang , et al., “Oxygen‐Promoted Low‐Temperature Hydrothermal Conversion of Chinese Medicinal Herbal Residues into Humic Acids,” Carbon Neutrality 4 (2025): 13–15.

[37] Q. Zhao , J. Zheng , X. Yan , et al., “Alkali‐Assisted Hydrothermal Preparation of Artificial Humic Acid From litchi Wood and Electrochemical Performance of Its Hydrochar,” Journal of Environmental Chemical Engineering 12 (2024): 113828, 10.1016/j.jece.2024.113828.

[38] S. A. Mondek , I. C. Constantino , O. P. Ferreira , et al., “Humic‐Like Substances Extracted From Hydrochar as an Additive for Foliar Fertilization in Tomato Plants,” ACS Omega 10, 40133–40145 (2025). 40949229 10.1021/acsomega.5c05053 PMC12423793

[39] Q. Lang , X. Guo , C. Wang , et al., “Characteristics and Phytotoxicity of Hydrochar‐derived Dissolved Organic Matter: Effects of Feedstock Type and Hydrothermal Temperature,” Journal of Environmental Sciences 149 (2025): 139–148, 10.1016/j.jes.2023.10.007. 39181629

[40] L. Soares Da Silva , I. C. Constantino , L. R. Bento , et al., “Humic Extracts From Hydrochar and Amazonian Anthrosol: Molecular Features and Metal Binding Properties Using EEM‐PARAFAC and 2D FTIR Correlation Analyses,” Chemosphere 256 (2020): 127110, 10.1016/j.chemosphere.2020.127110. 32464361

[41] D. Wang , X. Chen , J. Zhang , et al., “Alkaline‐thermal Synergistic Activation of Persulfate for Sawdust Hour‐level Humification to Prepare Fulvic‐Like‐acid Fertilizer,” Bioresource TechnolOGY 426 (2025): 132388, 10.1016/j.biortech.2025.132388. 40074092

[42] B. Yang , J. Zheng , Y. Sun , et al., “Air Heating‐alkaline Hydrothermal Production of Artificial Humic Acid: Rapid and Efficient Humification of Waste Biomass,” Journal of Environmental Chemical Engineering 13 (2025): 116770, 10.1016/j.jece.2025.116770.

[43] X. Peng , S. Gai , Z. Liu , K. Cheng , and F. Yang , “Effects of Fe^3+^ on Hydrothermal Humification of Agricultural Biomass,” Chemsuschem 17 (2024): 202301227, 10.1002/cssc.202301227. 37833827

[44] A. Volikov , H. Schneider , N. V. Tarakina , N. Marzban , M. Antonietti , and S. Filonenko , “Artificial Humic Substances as Sustainable Carriers for Manganese: Development of a Novel Bio‐based Microfertilizer,” Biofuel Research Journal 11 (2024): 2013–2024, 10.18331/BRJ2024.11.1.3.

[45] F. Deng , Z. Cao , Y. Luo , R. Wang , H. Shi , and D. Li , “Production of Artificial Humic Acid From Corn Straw Acid Hydrolysis Residue With Biogas Slurry Impregnation for Fertilizer Application,” Journal of Environmental Management 345 (2023): 118845, 10.1016/j.jenvman.2023.118845. 37619379

[46] T. Vitalii , M. Nader , V. Sarah , F. Svitlana , and A. Markus , “Chemical Insights Into the Base‐tuned Hydrothermal Treatment of Side Stream Biomasses,” Sustainable Energy & Fuels 7 (2023): 769–777.

[47] N. Jiao , Y. Zhu , H. Li , Y. Yu , Y. Xu , and J. Zhu , “Two‐Step Hydrothermal Pretreatments for Co‐Producing Xylooligosaccharides and Humic‐Like Acid From Vinegar Residue,” Fermentation 9 (2023): 589.

[48] Y.‐L. LI , X. CHEN , C.‐Q. ZHANG , et al., “Preparation and Structural Analysis of Humic Acid by Co‐Thermal Oxidation of Wheat Straw and Heilongjiang Lignite,” Journal of Fuel Chemistry and Technology 51 (2023): 145–154, 10.1016/S1872-5813(21)60033-1.

[49] S. Cai , W. Zhang , B. Yang , et al., “Alkali‐thermal Humification Treatment for Simultaneous Plant‐growth‐promoting Compounds Production and Antibiotic Removal From Lincomycin Fermentation Residues,” Chemical Engineering Journal 485 (2024): 149449, 10.1016/j.cej.2024.149449.

[50] Z. Liu , J. Su , Z. Yao , et al., “Effects of Decoupled Reaction Pressure From Temperature During Hydrothermal Humification of Lignocellulose,” Chemical Engineering Journal 511 (2025): 161911, 10.1016/j.cej.2025.161911.

[51] S. Yu , X. Dong , P. Zhao , et al., “Decoupled Temperature and Pressure Hydrothermal Synthesis of Carbon Sub‐micron Spheres From Cellulose,” Nature Communications 13 (2022): 3616, 10.1038/s41467-022-31352-x. PMC9232491 35750677

[52] S. Yu , M. Xie , Q. Li , Y. Zhang , and H. Zhou , “Evolution of Kraft Lignin During Hydrothermal Treatment Under Different Reaction Conditions,” Journal of the Energy Institute 103 (2022): 147–153, 10.1016/j.joei.2022.06.005.

[53] A. G. Demesa , A. Laari , I. Turunen , and M. Sillanpää , “Alkaline Partial Wet Oxidation of Lignin for the Production of Carboxylic Acids,” Chemical Engineering & Technology 38 (2015): 2270–2278, 10.1002/ceat.201400660.

[54] L. Wang , Y. Chi , K. Du , Z. Zhou , F. Wang , and Q. Huang , “Hydrothermal treatment of food waste for bio‐fertilizer production: Regulation of Humus Substances and Nutrient (N and P) in Hydrochar by Feedwater pH,” Waste and Biomass Valorization 14 (2023): 2767.

[55] Y. Jin , X. Zhang , Y. Yuan , Y. Lan , K. Cheng , and F. Yang , “Synthesis of Artificial Humic Acid‐urea Complex Improves Nitrogen Utilization,” Journal of Environmental Management 344 (2023): 118377, 10.1016/j.jenvman.2023.118377. 37348301

[56] Z.‐T. Hu , W. Huo , Y. Chen , et al., “Humic Substances Derived From Biomass Waste During Aerobic Composting and Hydrothermal Treatment: A Review,” Frontiers in Bioengineering and Biotechnology 10 (2022): 878686, 10.3389/fbioe.2022.878686. 35646832 PMC9133812

[57] Y. Shao , M. Bao , W. Huo , R. Ye , Y. Liu , and W. Lu , “Production of Artificial Humic Acid From Biomass Residues by a Non‐catalytic Hydrothermal Process,” Journal of Cleaner Production 335 (2022): 130302, 10.1016/j.jclepro.2021.130302.

[58] Y. Shao , J. Zhao , Y. Long , and W. Lu , “Two‐step Hydrothermal Conversion of Biomass Waste to Humic Acid Using Hydrochar as Intermediate,” Frontiers of Environmental Science & Engineering 17 (2023): 119, 10.1007/s11783-023-1719-8.

[59] Y. Shao , Z. Luo , M. Bao , et al., “Enhanced Production of Hydrothermal Humic Acid in a Two‐Step Hydrothermal Process With Acid Hydrothermal Solution Recycling,” Chemical Engineering Journal 474 (2023): 145634, 10.1016/j.cej.2023.145634.

[60] J. Su , Z. Liu , L. Zhao , et al., “A Sustainable Approach to Enhancing Humic Acid Production From Lignocellulose via Green Alum,” Journal of Environmental Chemical Engineering 13 (2025): 115156, 10.1016/j.jece.2024.115156.

[61] R. Nishimoto , S. Fukuchi , G. Qi , M. Fukushima , and T. Sato , “Effects of Surface Fe(III) Oxides in a Steel Slag on the Formation of Humic‐Like Dark‐Colored Polymers by the Polycondensation of Humic Precursors,” Colloids and Surfaces A: Physicochemical and engineering aspects 418 (2013): 117–123, 10.1016/j.colsurfa.2012.11.032.

[62] B. Zhang , J. Wang , Z. Xu , S. Wu , R. Luque , and H. Zhang , “Sewage Sludge Valorisation by Hydrothermal Carbonization: A New Method to Enhance Nitrogen Removal in Hydrochar Catalyzed With Ni–Mg–Al Layered Double Oxides,” Journal of Cleaner Production 386 (2023): 135880, 10.1016/j.jclepro.2023.135880.

[63] S. Sutradhar and P. Fatehi , “Latest Development in the Fabrication and Use of Lignin‐derived Humic Acid,” Biotechnology for Biofuels and Bioproducts 16 (2023): 38, 10.1186/s13068-023-02278-3. 36882875 PMC9989592

[64] Y. Shao , M. Bao , W. Huo , R. Ye , M. Ajmal , and W. Lu , “From Biomass to Humic Acid: Is There an Accelerated Way to Go?,” Chemical Engineering Journal 452 (2023): 139172, 10.1016/j.cej.2022.139172.

[65] Y. Zhu , Y. Cao , B. Fu , et al., “Waste Milk Humification Product Can be Used as a Slow Release Nano‐Fertilizer,” Nature Communications 15 (2024): 128, 10.1038/s41467-023-44422-5. PMC10761720 38167856

[66] F. Yang , S. Zhang , K. Cheng , and M. Antonietti , “A Hydrothermal Process to Turn Waste Biomass Into Artificial Fulvic and Humic Acids for Soil Remediation,” Science of The Total Environment 686 (2019): 1140–1151, 10.1016/j.scitotenv.2019.06.045. 31412510

[67] A. S. Amarasekara and F. Deng , “Single Reagent Treatment and Degradation of Switchgrass Using Iron(III)Chloride: The Effects on Hemicellulose, Cellulose and Lignin,” Biomass and Bioenergy 131 (2019): 105421, 10.1016/j.biombioe.2019.105421.

[68] F. Wang , Q. Liu , J. Chen , Z. Li , Y. Fu , and M. Qin , “Enhancement of Lignin Removal From Pre‐Hydrolysis Liquor for Saccharide Recovery via Horseradish Peroxidase Treatment in the Presence of Ca^2+^ ,” International Journal of Biological Macromolecules 163 (2020): 1989–1994, 10.1016/j.ijbiomac.2020.09.088. 32946940

[69] C. Li , G. Qian , R. Hong , X. Wang , and F. Qiu , “Experimental Research on Converting Biomass Waste Into Bio‐Fertilizer by Hydrothermal Treatment,” Journal of Agro‐Environment Science 23 (2004): 1119–1123.

[70] M. A. Lorenzo‐Santiago , J. Rodríguez‐Campos , R. Rendón‐Villalobos , E. García‐Hernández , A. A. Vallejo‐Cardona , and S. M. Contreras‐Ramos , “Thermal Treatment to Obtain 5‐Hydroxymethyl Furfural (5‐HMF), Furfural and Phenolic Compounds from Vinasse Waste from Agave,” Molecules 28 (2023): 1063. 36770727 10.3390/molecules28031063 PMC9919599

[71] E. Sarlaki , P. Ghofrani‐Isfahani , M. Ghorbani , et al., “Oxidation‐alkaline‐enhanced Abiotic Humification Valorizes Lignin‐rich Biogas Digestate Into Artificial Humic Acids,” Journal of Cleaner Production 435 (2024): 140409, 10.1016/j.jclepro.2023.140409.

[72] D. Mu , C. Wang , X. Geng , et al., “Effect of Maillard Reaction Based on Catechol Polymerization on the Conversion of Food Waste to Humus,” Chemosphere 353 (2024): 141560, 10.1016/j.chemosphere.2024.141560. 38417496

[73] Y. Zhao , K. Lu , H. Xu , L. Zhu , and S. Wang , “A Critical Review of Recent Advances in the Production of Furfural and 5‐hydroxymethylfurfural From Lignocellulosic Biomass Through Homogeneous Catalytic Hydrothermal Conversion,” Renewable and Sustainable Energy Reviews 139 (2021): 110706, 10.1016/j.rser.2021.110706.

[74] Y. Shao , Y. Geng , Z. Li , et al., “Unlocking the Potential of Humic Acid Production Through Oxygen‐assisted Hydrothermal Humification of Hydrochar,” Chemical Engineering Journal 472 (2023): 145098, 10.1016/j.cej.2023.145098.

[75] Y. Yan , X. Ma , W. Cao , et al., “Identifying the Reducing Capacity of Biomass Derived Hydrochar With Different Post‐treatment Methods,” Science of The Total Environment 643 (2018): 486–495, 10.1016/j.scitotenv.2018.06.232. 29945084

[76] X. Wang , A. Muhmood , R. Dong , and S. Wu , “Synthesis of Humic‐Like Acid From Biomass Pretreatment Liquor: Quantitative Appraisal of Electron Transferring Capacity and Metal‐binding Potential,” Journal of Cleaner Production 255 (2020): 120243, 10.1016/j.jclepro.2020.120243.

[77] C. Tang , Y. Li , J. Song , M. Antonietti , and F. Yang , “Artificial Humic Substances Improve Microbial Activity for Binding CO_2_ ,” iScience 24 (2021): 102647. 34466779 10.1016/j.isci.2021.102647 PMC8387571

[78] F. Yang , Y. Lan , R. Li , et al., “Anthropogenic Soil as an Environmental Material, as Exemplified With Improved Growth of Rice Seedlings,” Carbon Research 3 (2024): 46, 10.1007/s44246-024-00127-y.

[79] R. Ji , C. Liu , Q. Xu , et al., “Effect of Artificial Humic Acids Derived From Municipal Sludge on Plant Growth, Soil Fertility, and Dissolved Organic Matter,” Agriculture 14 (2024): 1946, 10.3390/agriculture14111946.

[80] S. Zhang , Q. Du , K. Cheng , M. Antonietti , and F. Yang , “Efficient Phosphorus Recycling and Heavy Metal Removal From Wastewater Sludge by a Novel Hydrothermal Humification‐Technique,” Chemical Engineering Journal 394 (2020): 124832, 10.1016/j.cej.2020.124832.

[81] Y. Zhao , Y. Hao , K. Cheng , et al., “Artificial Humic Acid Mediated Migration of Phosphorus in Soil: Experiment and Modelling,” Catena 238 (2024): 107896, 10.1016/j.catena.2024.107896.

[82] Z. Du , A. Hu , Q. Wang , et al., “Molecular Composition and Biotoxicity Effects of Dissolved Organic Matters in Sludge‐Based Carbon: Effects of Pyrolysis Temperature,” Journal of Hazardous Materials 424 (2022): 127346, 10.1016/j.jhazmat.2021.127346. 34601409

[83] S. D. Fatimah , A. Kuncaka , and R. Roto , “Characterization of Synthetic Humin From Solid Hydrolysate and Biochar From Hydrothermal Carbonization Products of Chicken Feather Waste,” Indonesian Journal of Chemistry 24 (2024): 1, 10.22146/ijc.78688.

[84] X. Li , Y. Zhi , M. Jia , et al., “Properties and Photosynthetic Promotion Mechanisms of Artificial Humic Acid Are Feedstock‐Dependent,” Carbon Research 3 (2024): 4, 10.1007/s44246-023-00085-x.

[85] P. Zhao , Z. Huang , Q. Ma , B. Zhang , and P. Wang , “Artificial Humic Acid Synthesized From Food Wastes: An Efficient and Recyclable Adsorbent of Pb (II) and Cd (II) From Aqueous Solution,” Environmental Technology & Innovation 27 (2022): 102399, 10.1016/j.eti.2022.102399.

[86] V. S. Santos , B. R. Moura , I. C. Constantino , et al., “Chelating Properties of Humic‐Like Substances Obtained From Process Water of Hydrothermal Carbonization,” Environmental Technology & Innovation 23 (2021): 101688, 10.1016/j.eti.2021.101688.

[87] S. J. Dos , L. G. Fregolente , A. B. Moreira , et al., “Humic‐Like Acids From Hydrochars: Study of the Metal Complexation Properties Compared With Humic Acids From Anthropogenic Soils Using PARAFAC and Time‐resolved Fluorescence,” Science of The Total Environment 722 (2020): 137815. 32179299 10.1016/j.scitotenv.2020.137815

[88] F. Yang , Q. Du , S. Long , and K. Cheng , “One‐step Fabrication of Artificial Humic Acid‐Functionalized Colloid‐Like Magnetic Biochar for Rapid Heavy Metal Removal,” Bioresource Technology 328 (2021): 124825, 10.1016/j.biortech.2021.124825. 33609885

[89] Q. Du , G. Li , S. Zhang , J. Song , Y. Zhao , and F. Yang , “High‐dispersion Zero‐valent Iron Particles Stabilized by Artificial Humic Acid for Lead Ion Removal,” Journal of Hazardous Materials 383 (2020): 121170, 10.1016/j.jhazmat.2019.121170. 31522068

[90] G. Ischia , N. Marzban , J. Schmidt , and A. Volikov , “Transitioning From Hydrothermal Carbonization to Humification for Producing Artificial Humic Substances,” Bioresource Technology 439 (2026): 133306, 10.1016/j.biortech.2025.133306. 40945799

[91] J. Mathew , A. Gopinath , and R. A. Vareed , “Spectroscopic Characterization of Humic Substances Isolated From Tropical Mangrove Sediments,” Arabian Journal of Geosciences 14 (2021): 668, 10.1007/s12517-021-06968-w.

[92] S. Ghobadian , G. Ischia , O. R. Romero , et al., “Hydrothermal Humification and Fulvification of Grass for Artificial Humic Substance Production and by‐Product Applications,” Environmental Technology & Innovation 41 (2026): 104836, 10.1016/j.eti.2026.104836.

[93] S. Celletti , A. Bergamo , V. Benedetti , et al., “Phytotoxicity of Hydrochars Obtained by Hydrothermal Carbonization of Manure‐based Digestate,” Journal of Environmental Management 280 (2021): 111635, 10.1016/j.jenvman.2020.111635. 33187784

[94] Q. Lang , B. Zhang , Y. Li , Z. Liu , and W. Jiao , “Formation and Toxicity of Polycyclic Aromatic Hydrocarbons During CaO Assisted Hydrothermal Carbonization of Swine Manure,” Waste Management 100 (2019): 84–90, 10.1016/j.wasman.2019.09.010. 31525676

[95] X.‐X. Peng , R. Li , C. Tang , et al., “Effects of Artificial Humic Acid on Decomposition of Returning Straw and Enhancement of Carbon Sequestration,” Applied Soil Ecology 203 (2024): 105619, 10.1016/j.apsoil.2024.105619.

[96] N. Marzban , J. A. Libra , V. S. Rotter , K. S. Ro , D. M. Paniagua , and S. Filonenko , “Changes in Selected Organic and Inorganic Compounds in the Hydrothermal Carbonization Process Liquid While in Storage,” ACS Omega 8 (2023): 4234–4243. 36743065 10.1021/acsomega.2c07419 PMC9893746

[97] S. Malghani , E. Jüschke , J. Baumert , et al., “Carbon Sequestration Potential of Hydrothermal Carbonization Char (hydrochar) in Two Contrasting Soils; Results of a 1‐year Field Study,” Biology and Fertility of Soils 51 (2015): 123–134, 10.1007/s00374-014-0980-1.

[98] Z. Jiang , S. Huang , and Z. Meng , “Long‐Term Effects of Biochar on the Hydraulic Properties of Soil: A Meta‐Analysis Based on 1–10 years Field Experiments,” Geoderma 458 (2025): 117318, 10.1016/j.geoderma.2025.117318.

[99] A. Gross , T. Bromm , S. Polifka , D. Fischer , and B. Glaser , “Long‐Term Biochar and Soil Organic Carbon Stability—Evidence from Field Experiments in Germany,” Science of The Total Environment 954 (2024): 176340, 10.1016/j.scitotenv.2024.176340. 39304170

[100] D. Busch , B. Glaser , M. Goss , and M. Goss , “Stability of Co‐Composted Hydrochar and Biochar Under Field Conditions in a Temperate Soil,” Soil Use Manage 31 (2015): 251–258.

[101] Tianji Agricultural Big Data Corn Stalk Prices in China, accessed October, 20, 2025, https://www.ymt.com.

[102] CEIC Data Industrial Electricity Prices in China, accessed October, 20, 2025, www.ceicdata.com.

[103] Prices of Chinese Herbal Medicine Residue, accessed October, 20, 2025, https://detail.1688.com.

[104] Trading Economics Labor Costs in China's Manufacturing Sector, accessed October, 20, 2025, https://zh.tradingeconomics.com.

[105] Chemicalbook Chemical Agents Market Prices, accessed October, 20, 2025, https://www.chemicalbook.com.

[106] U.S. Bureau of Labor Statistics Labor Costs in the U.S. Manufacturing Sector], accessed October, 20, 2025, https://www.bls.gov.

[107] United States Department of Agriculture Corn Stalk Prices in US, accessed October, 20, 2025, https://mymarketnews.ams.usda.gov.

[108] Businessanalytiq Chemical Agents Prices in US, accessed October, 20, 2025, https://businessanalytiq.com/procurementanalytics.

