# Supplementary material for: Hydrothermal Humification of Biomass for Circular Carbon Management in Sustainable Agro‐Ecosystems
Source: Adv Sci (Weinh). 2026 Jun 23;13(41):e75558. doi: 10.1002/advs.75558 (PMC13336092; doi:10.1002/advs.75558)
Supplement: Supplementary file 1 — Supporting File: advs75558‐sup‐0001‐SuppMat.docx. [file ADVS-13-e75558-s001.docx]

**Supplementary Materials**

**Table S1** **Engineering HTH process emission**

unit: kg CO₂e per kg biomass

| **Scenarios** | **Heating** | **Grinding** | **Separation** | **Chemicals** | **Water** | **Total** |
| --- | --- | --- | --- | --- | --- | --- |
| **G1** | **1.84** | 0.022 | 0.011 | 0.066 | 0.019 | **1.96** |
| **G2** | **1.03** | 0.022 | 0.011 | 0.068 | 0.010 | **1.14** |
| **G3** | **0.03** | 0.022 | 0.011 | 0.060 | 0.008 | **0.13** |
| **G4** | **0.39** | 0.022 | 0.011 | 0.031 | 0.017 | **0.48** |
| **G5** | **0.39** | 0.022 | 0.011 | 0.032 | 0.016 | **0.48** |
| **G6** | **3.10** | 0.022 | 0.011 | 0.072 | 0.025 | **3.23** |
| **G7** | **1.53** | 0.022 | 0.011 | 0.050 | 0.017 | **1.63** |
| **G8** | **0.00** | 0.022 | 0.011 | 0.005 | 0.013 | **0.051** |
| **G9** | **0.00** | 0.022 | 0.011 | 0.003 | 0.013 | **0.049** |
| **G10** | **3.90** | 0.022 | 0.011 | 0.091 | 0.026 | **4.05** |
| **G11** | **0.10** | 0.022 | 0.011 | 0.056 | 0.017 | **0.206** |

Industrial energy intensities were estimated from commercial equipment specifications (0.0367 kWh kg⁻¹ for grinding, 0.18 kWh kg⁻¹ for heating, and 0.02 kWh kg⁻¹ for centrifugation), combined with the regional electricity carbon intensity (0.612 kg CO₂e kWh⁻¹). Chemical emissions were calculated from activity-specific emission factors (kg CO₂e/kg, HCl: 1.38, NaOH: 1.03, KOH: 1.90, and H₃PO₄: 5.30), and water emissions used a factor of 8.02×10⁻⁴ kg CO₂e per kg water. The resulting industrial-scale process emissions ranged from 0.049 to 4.05 kg CO₂e per kg feedstock, depending on reaction conditions and chemical dosage.

**Table S2** **Hydrochar yield, composition, and carbon distribution (hydrochar basis)**

| **Scenarios** | $\boldsymbol{Y}_{\text{hc}}$**(kg/kg biomass)** | $\boldsymbol{w}_{\text{C,hc}}$ **(%)** | $\boldsymbol{f}_{\text{HHA}}$ **(kg/kg hydrochar)** | $\boldsymbol{w}_{\boldsymbol{C, HHA}}$ **(%)** | $\boldsymbol{f}_{\text{non}}$ **(kg/kg hydrochar)** | $\boldsymbol{w}_{\boldsymbol{C,}\boldsymbol{non}}$ **(%)** |
| --- | --- | --- | --- | --- | --- | --- |
| G1 | 29.6 | 59.6 | 0.197 | 58.6 | 0.803 | **47.86** |
| G2 | 32.1 | 50.0 | 0.233 | 50.0 | 0.767 | **38.35** |
| G3 | 33.1 | 45.0 | 0.235 | 44.4 | 0.765 | **34.43** |
| G4 | 36.0 | 55.0 | 0.109 | 65.0 | 0.891 | **49.03** |
| G5 | 34.9 | 58.2 | 0.068 | 75.1 | 0.932 | **54.26** |
| G6 | N.A. | N.A. | N.A. | N.A. | N.A. | N.A. |
| G7 | N.A. | N.A. | N.A. | N.A. | N.A. | N.A. |
| G8 | 58.0 | 50.0 | 0.305 | 56.5 | 0.695 | **34.76** |
| G9 | 52.6 | 55.5 | 0.353 | 56 | 0.647 | **35.92** |
| G10 | 52.6 | 46.4 | 0.313 | 56.5 | 0.688 | **31.90** |
| G11 | 6.80 | 29.7 | 0.492 | 56.0 | 0.508 | **15.09** |

**Hydrochar yield:**

$Y_{\text{hc}}$=$m_{\text{hy}\text{drochar}}$ / $m_{\text{biomass}}$ (ES1)

**HHA and non-HHA mass fractions in hydrochar:**

$f_{\text{HHA}}= m_{\text{HHA}}$ / $m_{\text{h}\text{c}}$ (ES2)

$f_{\text{non}}=1-f_{\text{HHA}}$ (ES3)

**Carbon content of non-HHA fraction:**

$C_{\text{HHA}}=m_{\text{HHA}}\cdot w_{\text{C,HHA}}$ (ES4)

$C_{\text{non}}={C_{\text{hc}}-C}_{\text{HHA}}$ (ES5)

where, $Y_{\text{hc}}$ represents the hydrochar yield; $m_{\text{HHA}}$ and $m_{\text{h}\text{c}}$ represent the mass of HHA and hydrochar, respectively; $f_{\text{HHA}}$ and $f_{\text{non}}$represents the mass fractions of HHA and non-HHA fraction in hydrochar, respectively; $w_{\text{C,HHA}}$ represents C content of HHA; $C_{\text{hc}}$, $C_{\text{HHA}}$ and $C_{\text{non}}$ represent C mass in hydrochar, HHA and non-HHA fraction, respectively.

**Table S3 Carbon and stable carbon in HHA and non-HHA fractions of hydrochar (on a biomass basis)**

| **Scenario** | $\boldsymbol{m}_{\text{HHA}}$ **(kg/kg)** | $\boldsymbol{m}_{\text{non}}$ **(kg/kg)** | $\boldsymbol{C}_{\text{HHA}}$ **(kg C)** | $\boldsymbol{C}_{\text{non}}$ **(kg C)** | $\boldsymbol{C}_{\text{stab,HHA}}$ **(kg C)** | $\boldsymbol{C}_{\text{stab,non}}$ **(kg C)** |
| --- | --- | --- | --- | --- | --- | --- |
| G1 | 0.0583 | 0.2377 | 0.0342 | 0.1422 | 0.0120 | 0.0850 |
| G2 | 0.0748 | 0.2462 | 0.0374 | 0.1231 | 0.0131 | 0.0739 |
| G3 | 0.0778 | 0.2532 | 0.0345 | 0.1144 | 0.0121 | 0.0683 |
| G4 | 0.0391 | 0.3209 | 0.0254 | 0.1726 | 0.0089 | 0.1059 |
| G5 | 0.0237 | 0.3258 | 0.0178 | 0.1856 | 0.0062 | 0.1138 |
| G6 | 0 | 0 | 0 | 0 | 0 | 0 |
| G7 | 0 | 0 | 0 | 0 | 0 | 0 |
| G8 | 0.1768 | 0.4032 | 0.0999 | 0.1901 | 0.0350 | 0.1209 |
| G9 | 0.1856 | 0.3404 | 0.1039 | 0.1880 | 0.0364 | 0.1134 |
| G10 | 0.1644 | 0.3616 | 0.0836 | 0.1605 | 0.0292 | 0.1007 |
| G11 | 0.0335 | 0.0345 | 0.0192 | 0.0010 | 0.0067 | 0.0062 |

**HHA mass on biomass basis**

$m_{\mathrm{HHA}}=Y_{\mathrm{hc}}\times f_{\mathrm{HHA}}$ (ES6)

**Non-HHA mass on biomass basis**

$m_{\mathrm{non}}=Y_{\mathrm{hc}}\times f_{\mathrm{non}}$ (ES7)

**Total carbon in hydrochar**

$C_{\mathrm{hc},\mathrm{total}}=Y_{\mathrm{hc}}\times w_{C,\mathrm{hc}}$ (ES8)

**Carbon mass in HHA**

$C_{\mathrm{HHA}}=m_{\mathrm{HHA}}\times w_{C,\mathrm{HHA}}$ (ES9)

**Carbon mass in non-HHA fraction**

$C_{\mathrm{non}}=C_{\mathrm{hc},\mathrm{total}}-C_{\mathrm{HHA}}$ (ES10)

**Stable carbon mass in HHA:**

$C_{\text{stab,HHA}}=C_{\text{HHA}}\times\alpha_{HHA}$ (ES11)

**Stable carbon mass in non-HHA:**

$C_{\text{stab,non}}=C_{\text{non}}\times\alpha_{non}$ (ES12)

where, and $C_{\mathrm{non}}$are carbon masses in HHA and non-HHA fractions of hydrochar, respectively, expressed as kg C per kg biomass; and $\alpha_{\mathrm{non}}$are stability coefficients for HHA and non-HHA carbon, respectively. In this study, $\alpha_{\mathrm{HHA}}=0.35$and $\alpha_{\mathrm{non}}=0.60$.

**Table S4 Carbon sequestration benefits and net emissions**

Unit: kg CO₂e per kg biomass

| **Scenario** | **Process emission** | **Solid HHA** $\mathbf{C}_{\text{store}}$ | **Liquid-HHA** $\mathbf{C}_{\text{store}}$ | **Non-HHA** $\mathbf{C}_{\text{store}}$ | **Total soil C-store** | **Total benefit*** | **Net emission** |
| --- | --- | --- | --- | --- | --- | --- | --- |
| G1 | 1.963 | 0.044 | 0.000 | 0.312 | 0.356 | 0.448 | +1.515 |
| G2 | 1.140 | 0.048 | 0.000 | 0.271 | 0.319 | 0.411 | +0.729 |
| G3 | 0.133 | 0.044 | 0.000 | 0.251 | 0.295 | 0.387 | −0.254 |
| G4 | 0.481 | 0.033 | 0.063 | 0.388 | 0.484 | 0.576 | −0.095 |
| G5 | 0.480 | 0.023 | 0.043 | 0.417 | 0.483 | 0.575 | −0.095 |
| G6 | 3.228 | 0.000 | 0.189 | 0.000 | 0.189 | 0.281 | +2.947 |
| G7 | 1.629 | 0.000 | 0.326 | 0.000 | 0.326 | 0.418 | +1.211 |
| G8 | 0.051 | 0.128 | 0.000 | 0.444 | 0.572 | 0.664 | −0.613 |
| G9 | 0.049 | 0.134 | 0.000 | 0.416 | 0.549 | 0.641 | −0.592 |
| G10 | 4.050 | 0.107 | 0.000 | 0.369 | 0.476 | 0.568 | +3.482 |
| G11 | 0.206 | 0.025 | 0.000 | 0.023 | 0.047 | 0.139 | +0.067 |

*Total benefit = Soil carbon sequestration + Avoided burning (0.092 kg CO₂e·kg⁻¹)

**Total emissions reduction:**

$\text{SoilC}_{CO_{2}e}=(C_{\text{H}\text{H}\text{A,solid}\text{ }}\times0.35+C_{\text{H}\text{H}\text{A,liq}}\times0.35+C_{\text{non}}\times0.60)\times{44}/{12}$ (ES13)

**Total benefit**

$\text{Total benefit}=\text{SoilC}_{CO_{2}e}+E_{\text{avoid}}$ (ES14)

**Net emissions:**

$\text{Net}=E_{\mathrm{proc}}-(E_{\mathrm{avoid}}+\text{SoilC}_{CO_{2}e})$ (ES15)

where, $C_{\text{H}\text{H}\text{A,solid}}$, $C_{\mathrm{HHA},\mathrm{liq}}$, and $C_{\mathrm{non}}$ represent the carbon mass of the solid-phase HHA, liquid-phase HHA, and non-HHA fraction, respectively; $E_{\mathrm{proc}}$ and $E_{\mathrm{avoid}}$denote process emissions and avoided emissions from biomass burning, respectively, with $E_{\mathrm{avoid}}$ fixed at 0.092 kg CO₂e per kg biomass; $\mathrm{SoilC}_{CO_{2}e}$ represents soil carbon sequestration; Net denotes net greenhouse gas emissions.

**Table S5** **Overall Stability Sensitivity and Carbon Neutrality Threshold** $\boldsymbol{S}_{\text{crit}}$

| **Scenario** | $\mathbf{SoilC}_{\boldsymbol{C}\boldsymbol{O}_{\boldsymbol{2}}\boldsymbol{e,}\text{ref}}$ **(kg CO₂e/kg)** | ***S_crit_*** |
| --- | --- | --- |
| G1 | 0.356 | 5.26 |
| G2 | 0.319 | 3.29 |
| G3 | 0.295 | 0.14 |
| G4 | 0.484 | 0.80 |
| G5 | 0.483 | 0.80 |
| G6 | 0.189 | 16.6 |
| G7 | 0.326 | 4.71 |
| G8 | 0.572 | ＜0 |
| G9 | 0.549 | ＜0 |
| G10 | 0.476 | 8.31 |
| G11 | 0.047 | 2.42 |

**Total Soil Carbon Sequestration:**

$$\text{SoilC}_{CO_{2}e}(s)=S\times\text{SoilC}_{CO_{2}e,\mathrm{ref}}$$

**Carbon-neutral conditions (net emission is 0):**

$$s_{\text{crit}}=\frac{E_{\mathrm{proc}}-E_{\mathrm{avoid}}}{\text{SoilC}_{CO_{2}e,\mathrm{ref}}}$$

where $\mathrm{SoilC}_{CO_{2}e,\text{ref}}$represents the reference soil carbon sequestration under baseline stability assumptions, and $s$is a scaling factor applied uniformly to all carbon pools. $s_{\text{crit}}$denotes the minimum stability factor required to achieve net-zero emissions.

**Table S6 Sensitivity of net emissions to carbon stabilization factor**

| **Scenario** | $\boldsymbol{\alpha}_{\mathbf{solid}}$ | $\mathbf{SoilC}_{\mathbf{with}}$(kg CO₂e·kg/ biomass) | $\mathbf{Net}_{\mathbf{with}}$ (kg CO₂e·kg/ biomass) |
| --- | --- | --- | --- |
| G1 | 0.5 | 0.322 | 1.549 |
| G1 | 0.7 | 0.451 | 1.420 |
| G1 | 0.9 | 0.580 | 1.291 |
| G2 | 0.5 | 0.294 | 0.754 |
| G2 | 0.7 | 0.412 | 0.636 |
| G2 | 0.9 | 0.530 | 0.518 |
| G3 | 0.5 | 0.272 | −0.231 |
| G3 | 0.7 | 0.381 | −0.340 |
| G3 | 0.9 | 0.490 | −0.449 |
| G4 | 0.5 | 0.424 | −0.035 |
| G4 | 0.7 | 0.572 | −0.183 |
| G4 | 0.9 | 0.720 | −0.331 |
| G5 | 0.5 | 0.417 | −0.029 |
| G5 | 0.7 | 0.567 | −0.179 |
| G5 | 0.9 | 0.717 | −0.329 |
| G6 | 0.5 | 0.099 | 3.041 |
| G6 | 0.7 | 0.139 | 3.000 |
| G6 | 0.9 | 0.179 | 2.959 |
| G7 | 0.5 | 0.279 | 1.258 |
| G7 | 0.7 | 0.391 | 1.146 |
| G7 | 0.9 | 0.503 | 1.034 |
| G8 | 0.5 | 0.458 | −0.315 |
| G8 | 0.7 | 0.641 | −0.498 |
| G8 | 0.9 | 0.825 | −0.682 |
| G9 | 0.5 | 0.439 | −0.298 |
| G9 | 0.7 | 0.615 | −0.474 |
| G9 | 0.9 | 0.791 | −0.651 |
| G10 | 0.5 | 0.381 | 3.577 |
| G10 | 0.7 | 0.534 | 3.424 |
| G10 | 0.9 | 0.687 | 3.271 |
| G11 | 0.5 | 0.027 | 0.087 |
| G11 | 0.7 | 0.037 | 0.077 |
| G11 | 0.9 | 0.048 | 0.066 |

where, $\alpha_{\mathrm{solid}}$ represents the stabilization coefficient of the solid-phase carbon pool; $\mathrm{SoilC}_{\mathrm{with}}$and $\mathrm{Net}_{\mathrm{with}}$denote the resulting soil carbon sequestration and net CO₂-equivalent emissions, respectively. The solid carbon pool includes both solid-phase HHA and non-HHA fractions in hydrochar. Sensitivity analysis was conducted over $\alpha_{\mathrm{solid}}=0.5\text{–}0.9$, while the stabilization coefficient for liquid-phase HHA was fixed at $\alpha_{\mathrm{liquid}}=0.3$.
